# HNRNPL induced circFAM13B increased bladder cancer immunotherapy sensitivity via inhibiting glycolysis through IGF2BP1/PKM2 pathway

**DOI:** 10.1186/s13046-023-02614-3

**Published:** 2023-02-06

**Authors:** Jiancheng Lv, Kai Li, Hao Yu, Jie Han, Juntao Zhuang, Ruixi Yu, Yidong Cheng, Qiang Song, Kexin Bai, Qiang Cao, Haiwei Yang, Xiao Yang, Qiang Lu

**Affiliations:** Department of Urology, The First Affiliated Hospital of Nanjing Medical University, No. 300 Guangzhou Road, Nanjing, 210029 China

**Keywords:** circFAM13B, Bladder cancer, IGF2BP1, PKM2, Glycolysis, Immunosensitivity

## Abstract

**Background:**

The response rate to immunotherapy in patients with bladder cancer (BCa) remains relatively low. Considering the stable existence and important functions in tumour metabolism, the role of circRNAs in regulating immune escape and immunotherapy sensitivity is receiving increasing attention.

**Methods:**

Circular RNA (circRNA) sequencing was performed on five pairs of BCa samples, and circFAM13B (hsa_circ_0001535) was screened out because of its remarkably low expression in BCa. Further mRNA sequencing was conducted, and the association of circFAM13B with glycolysis process and CD8^+^ T cell activation was confirmed. The functions of circFAM13B were verified by proliferation assays, glycolysis assays, BCa cells-CD8^+^ T cell co-culture assays and tumorigenesis experiment among human immune reconstitution NOG mice. Bioinformatic analysis, RNA–protein pull down, mass spectrometry, RNA immunoprecipitation, luciferase reporter assay and fluorescence in situ hybridization were performed to validate the HNRNPL/circFAM13B/IGF2BP1/PKM2 cascade.

**Results:**

Low expression of circFAM13B was observed in BCa, and it was positively associated with lower tumour stage and better prognosis among patients with BCa. The function of CD8^+^ T cells was promoted by circFAM13B, and it could attenuate the glycolysis of BCa cells and reverse the acidic tumour microenvironment (TME). The production of granzyme B and IFN-γ was improved, and the immunotherapy (PD-1 antibodies) sensitivity was facilitated by the inhibition of acidic TME. Mechanistically, circFAM13B was competitively bound to the KH3-4 domains of IGF2BP1 and subsequently reduced the binding of IGF2BP1 and PKM2 3’UTR. Thus, the stability of the PKM2 mRNA decreased, and glycolysis-induced acidic TME was inhibited. The generation of circFAM13B was explored by confirming whether heterogeneous nuclear ribonucleoprotein L (HNRNPL) could promote circFAM13B formation via pre-mRNA back-splicing.

**Conclusions:**

HNRNPL-induced circFAM13B could repress immune evasion and enhance immunotherapy sensitivity by inhibiting glycolysis and acidic TME in BCa through the novel circFAM13B/IGF2BP1/PKM2 cascade. Therefore, circFAM13B can be used as a biomarker for guiding the immunotherapy among patients with BCa.

**Supplementary Information:**

The online version contains supplementary material available at 10.1186/s13046-023-02614-3.

## Background

Bladder cancer (BCa) is one of the most common malignant tumours in the genitourinary system [1]. It could be classified into non-muscle invasive BCa (NMIBC), which is prone to relapse, and muscle invasive BCa (MIBC), which is prone to metastasis [2, 3]. In recent years, the safety and efficacy of immune checkpoint inhibitors (ICIs) that target PD-1/PD-L1 have been verified in many cancer types, which is an important breakthrough in BCa comprehensive treatment [4, 5]. However, the response rate of immunotherapy in BCa patients is only about 25% [6]. Therefore, the mechanism of immune evasion and immunotherapy resistance needs to be explored.

Aerobic glycolysis, which is also termed as Warburg effect, is among the most important mechanisms in tumorigenesis and development [7]. During glycolysis, the tumour cells consume glucose, produce lactic acid and release it into the tumour microenvironment (TME), thus reducing the pH value of the TME [8]. The acidic TME induced by tumour glycolysis can directly inhibit the cytotoxicity and proliferation of CD8^+^ T cells, which could actively promote immune escape and inhibit immunotherapy sensitivity [9]. Immunosuppressive cell types such as regulatory T cells (Treg) and myeloid-derived suppressor cells (MDSC) can also be recruited and induced by acidic TME to further inhibit the anti-tumour immune response [10]. By altering the function of immune cells represented by CD8 + T cells, acidic TME has the potential to promote tumour immune evasion and inhibit immunotherapy sensitivity [11]. Pyruvate kinase muscle isozyme M2 (PKM2) is a key enzyme in the last step of glycolysis, and it is directly involved in the formation of lactic acid and acidic TME [12, 13]. PKM2 could promote the progression of different cancers, including BCa [14–17]. PKM2 could also inhibit the immune response by promoting the glycolysis and formation of acidic TME [15]. Therefore, the regulation of PKM2 is among the key mechanisms that affect acidic TME and interfere with immune escape and immunotherapy sensitivity [15].

Circular RNA (circRNA), which has a more stable covalent closed-loop structure than linear RNA, has important potential to influence the acidic TME and immunosuppressive state by regulating tumour glycolysis [18–20]. CircRNA can perform functions in different mechanisms, including acting as microRNAs (miRNAs) sponges, binding to proteins to regulate gene transcription or translation and acting as peptides or protein translation templates [21]. For circRNAs that could neither be recognised by miRNAs nor translated, the core mechanism to perform a function could be binding to RNA binding proteins (RBPs) [22]. circRNA binds and regulates the function of insulin-like growth factor-2 mRNA-binding protein 1 (IGF2BP1), which could recognise the N6-methyladenosine (m6A) modification and bind to mRNA to maintain its stability [23, 24]. circPTPRA could bind and block the function of IGF2BP1, thus further suppressing the progression of BCa [24]. IGF2BP1 participates in the glycolysis of tumour cells and plays a significant role in the formation of acidic TME and the occurrence of immune escape [25, 26]. In the present study, circFAM13B (hsa_circ_0001535) was identified by high-throughput circRNA sequencing. The results confirmed that circFAM13B could inhibit the proliferation, immune escape, and increase in the immunotherapy sensitivity of BCa by attenuating the glycolysis process via a novel IGF2BP1/PKM2 cascade.

The downstream of circFAM13B was explored, and its formation was studied. As a pro-splicing factor of RNA, heterogeneous nuclear ribonucleoprotein L (HNRNPL) can promote the back-splicing of circRNA by binding to the upstream and downstream intron sequences [27]. For instance, the formation of circANKRD42 and circARHGAP35 is inseparable from the assistance of HNRNPL [28, 29]. The formation of circFAM13B was regulated by HNRNPL. The results of this study revealed a new mechanism of immunotherapy sensitisation and identified a novel potential target for BCa treatment.

## Methods

### Human tissue specimens and cell lines

We obtained 72 pairs of BCa tissues from patients undergoing radical BCa surgery at the First Affiliated Hospital of Nanjing Medical University between 2013 and 2021. The acquisition and utilization of the tissues have been approved by the ethics board of hospital, and informed consent was obtained from the patients. Subsequently, we analysed the clinical-pathological features of 72 patients with BCa. According to the follow-up data, patient survival ranged from 1 month to 62.5 months. Follow-up data were recorded from the end of surgery to the onset of disease progression or recurrence. The methodology used in this study was fully compliant with the guidelines of the Declaration of Helsinki. Seven BCa cell lines (BIU87, T24, RT4, 5637, 253 J, UMUC3, and J82) and a normal bladder epithelial immortalised cell line (SV-HUC) were obtained from the Type Culture Collection of the Chinese Academy of Sciences (Shanghai, China).

### Cell culture and transfection

T24, UMUC3 and HEK 293 T cells were cultured with Dulbecco’s modified eagle’s medium (Gibco, USA) containing 10% foetal bovine serum (FBS, BI, Israel) in a humidified incubator at 37 °C with 5% CO_2_.

CircFAM13B knockdown and overexpression lentivirus vectors were acquired from HANBIO (HANBIO, Shanghai, China). T24 or UMUC3 cells were transfected with circFAM13B knockdown (sh circFAM13B-1 and sh circFAM13B-2), negative control (sh NC), circFAM13B overexpression (circFAM13B) and relative control (vector) lentivirus when the cells reached 50% confluence. The transfected cells were screened twice by using puromycin (4 and 8 μg/mL). Overexpression plasmids and small interfering RNAs (siRNAs) of IGF2BP1, HNRNPL and PKM2 were obtained from GenePharma Co. (Shanghai, China). T24 and UMUC3 cells were transfected with the previously mentioned samples by using the Lipofectamine 3000 kit (Invitrogen, USA) according to the guideline. The siRNAs/shRNAs used in this research were listed in Additional table S1.

### RNA extraction and quantitative real time-PCR (qRT-PCR)

Total RNA was extracted from the BCa tissues or cells by using TRIzol reagent (Invitrogen, USA). Then, the RNA was reverse-transcribed into cDNA by using HiScript II reagent (Vazyme, China). qRT-PCR experiments were conducted to measure circRNA or mRNA level with SYBR pre-mix kit (Vazyme, Nanjing, China). The data were analyzed using the StepOne Plus real-time PCR system (Applied Biosystems, USA) or LightCycler 480 (Roche, USA). The primers used in this study were acquired from TsingKe (TsingKe, Nanjing, China) and listed in Additional table S2.

### Protein isolation and western blot

Tissue and cell proteins were extracted using RIPA buffer (Sigma, USA). The concentration of proteins was measured using the bicinchoninic acid (BCA) kit (Beyotime, China). Different proteins were isolated by SDS-PAGE, and then transferred onto polyvinylidene fluoride membranes. After blocking with 5% skim milk, membranes were incubated with indicated primary antibodies (Abcam, USA) and secondary antibodies (Protech, USA) sequentially. After washing with several times, different proteins were identified using chemiluminescence (Bio-Rad, USA) and analysed with Image Lab (Bio-Rad, USA).

### RNase R treatment assay

The total RNA of T24 or UMUC3 cells was treated with RNase R (0.2 μl/μg, Epicenter) for 30 min at 37 °C. Then, the expression of circFAM13B and FAM13B mRNA was measured using qRT-PCR.

### Actinomycin D treatment and RNA stability assay

T24 or UMUC3 cells were treated with 1 μg/ml actinomycin D (Abcam, UK) at indicated times (0, 2, 4, 6, 8, and 10 h). Then, the total RNA of these cells was extracted, and the expression levels of circFAM13B, FAM13B, and PKM2 were determined using qRT-PCR.

### RNA–protein pulldown, silver staining and mass spectrometry

RNA–protein pulldown assays were carried out using the Pierce Magnetic RNA–Protein Pull-Down kit (Thermo Fisher Scientific) based on the manufacturer’s protocol. Biotin-labelled probes, which could bind the back-splice junction sites or the flanking intron sequence of circFAM13B, were obtained from Keygentec (Nanjing, China). Cells were lysed with IP lysis buffer and incubated with indicated probe at room temperature for 4 h. The proteins isolated by probes were identified via Western blot analysis. Fast silver staining kit (Beyotime, Shanghai, China) was used to perform the silver staining assay according to the protocol. Further mass spectrometry analysis was performed using Keygentec (Nanjing, China). Proteins were identified and quantified using the Proteome Discoverer Software 2.4 (Thermo Fisher Scientific).

### Immunofluorescence assay and fluorescence in situ hybridization (IF-FISH)

BCa cells were cultured in a confocal dish and fixed with 4% paraformaldehyde. Once fixed, cells were permeabilized with 0.1% Triton X-100 (Solarbio, Beijing, China). After blocking with 3% bovine serum albumin, the cells were incubated with indicated primary antibodies (Abcam, USA) and secondary fluorescent antibodies (Protech, USA) sequentially. Cy3-labeled circFAM13B probes were acquired from GenePharma (Shanghai, China). The cells were stained by incubation with the circFAM13 probe by using the RNA-FISH kit (Genepharma, Shanghai, China) according to the established instruction. After staining with DAPI for 30 min, cell images were obtained using the Zeiss LSM880 NLO confocal microscope system (Leica Microsystems, Germany).

### Dual-luciferase reporter assay

The binding of IGF2BP1 and PKM2 3’ UTR was assessed by synthesising the PKM2 3’ UTR wild type (WT) luciferase reporter plasmid and binding motif-mutated luciferase reporter plasmid (WT) by GenePharma (Shanghai, China). Then, circFAM13B overexpression or relative control cells were co-transfected with IGF2BP1 overexpression plasmid and the above WT or MT reporter plasmids by using Lipofectamine 3000 (Invitrogen, USA). After 24 h of transfection, firefly and Renilla luciferase activities were acquired consistently by using the dual-luciferase reporter assay system (Promega, USA).

### RNA immunoprecipitation (RIP)

Cells were harvested and lysed by using the IP lysis buffer. The Magna RIP RNA binding protein immunoprecipitation kit (Millipore, USA) was used to perform the RIP assays according to the established protocol. Antibodies specific to IGF2BP1 (Abcam, USA), FLAG (CST, USA), HNRNPL (Protech, USA), and control IgG (Millipore, USA) were used to precipitate the RNA. Input and co-immunoprecipitated RNAs were extracted using TRIzol reagent (Invitrogen, USA). The specified RNA was measured by qRT-PCR and normalised to the input.

### Cell proliferation and cloning formation assays

The proliferation ability of BCa cells was assessed by seeding 2,000 T24 or UMUC3 cells in each well of 96-well plates. Cell counting kit-8 (CCK8; Dojindo, Japan) assays were performed to measure the cell viability at different times (24, 48, 72, and 96 h). A microplate reader (Tecan, Switzerland) was used to record the absorbance values at 450 nm.

Approximately 1,000 T24 or UMUC3 cells were seeded in each well of six-well plates. After 14 days, the cells were fixed with 4% paraformaldehyde and stained with 0.1% crystal violet. The cell colonies were visualised and calculated using Image J software (NIH, USA).

Lactic acid, glucose and adenosine triphosphate (ATP) detection

The consumption of glucose, production of lactic acid and synthesis of intracellular ATP were detected using the glucose, lactic acid and ATP assay kits (Jiancheng Corporation, Nanjing, China), respectively, according to the established instruction.

### CD8 + T cell culture and CD8 + T cell–mediated BCa cell killing assay

Peripheral blood mononuclear cells (PBMCs) in the peripheral blood of healthy person were isolated using PBMC separation reagent (FACs, Nanjing, China) and cultured in RPMI-1640 medium (Gibco, USA). CD8^+^ T cells that are bound to CD8 microbeads (Miltenyi, Germany) were sorted out from PBMCs by magnetic separation. The positive rate of cell sorting was determined by flow cytometry by using FITC-CD3 antibodies (Biolegend, USA) and APC-CD8 antibodies (Biolegend, USA). CD8^+^ T cells were cultured in RPMI-1640 medium and activated by adding CD3 antibodies (2 μg/mL; Invitrogen, USA), CD28 antibodies (1 μg/mL; Invitrogen, USA), and interleukin 2 (IL-2, 5 ng/mL; R&D Systems, USA). After 72 h, activated CD8^+^ T cells were co-cultured with BCa cells at a ratio of 1:2.5 for 48 h. Then, a proper amount of medium was collected from the co-culture system to detect the content of granzyme B and IFN-γ by using the ELISA kit (FACs, Nanjing, China). The killing ability and immunotherapy sensitivity were investigated by co-culturing the activated CD8 + T cells with BCa cells at a ratio of 2:1 for 72 h. We divided the BCa cells into three groups. The first group was the control group, which was not co-cultured with CD8^+^ T cells. The second group was co-cultured with CD8^+^ T cells proportionally. The third group was co-cultured with CD8^+^ T cells, in which the medium was added with PD1 antibodies (Bio X cell, USA) at a concentration of 10 μg/mL. After removing the debris and T cells by washing with PBS for several times, living BCa cells were quantified using a spectrometer at 570 OD. Then, BCa cells were fixed with 4% paraformaldehyde, and then stained with 0.1% crystal violet. The BCa cells were visualised using the Image J software (NIH, USA).

### Humanised NOG mice generation and xenograft model

NOG (NOD-scid IL2Rg^null^) mice were obtained from Shanghai Charles River Co. As a mouse with severe immune deficiency, its T and B cells were lost, NK cell function was lost, macrophage function was decreased, and the immune regulation ability was decreased. The human immune system in NOG mice (5 weeks old, female, five mice per group) was reconstructed by transfecting 10^7^ PBMCs from healthy human peripheral blood into the mice via the tail vein. Meanwhile, 10^7^ T24 cells (circFAM13B or vector) were inoculated into the axilla of NOG mice. Blood samples were obtained from the orbit of the mice, and the proportion of human CD45-positive PBMCs was determined by flow cytometry by using PE-CD45 antibodies (Biolegend, USA) once a week. If the positive rate exceeded 50%, the construction of HuNOG mice was considered successful. When the tumour volume reached 100 mm^3^, HuNOG mice in the experimental groups were injected with PD1-antidies (5 μg/g) intraperitoneally every other day and maintained for 2 weeks, while the HuNOG mice in control groups were injected with IgG antibodies. Since the injection of PD1 antibodies, tumour size was measured every 3 days. After a month, all HuNOG mice were euthanised, and the tumour specimens were removed for further weighing and immunohistochemical testing. Ethical approval of animal assays was obtained from the Animal Ethics Board of Nanjing Medical University.

### Immunohistochemistry (IHC)

Paraffin-embedded HuNOG mice tumours were spliced into 4 μm sections and mounted on slides. Then, the specimens were rehydrated by treatment with different grades of ethanol. Microwave heating was used to isolate the antigens. After dipping in 3% H_2_O_2_ for 10 min, the slides were treated with Ki-67 (Proteintech, USA), PKM2 (Abcam, USA), CD8 (Proteintech, USA) and CD3 antibodies (Proteintech, USA) at 4 °C overnight. Afterward, HRP-conjugated antibody was used to treat the slides at room temperature for 30 min. The images were viewed and recorded under a microscope.

### RNA sequencing

Total RNA was extracted from circFAM13B overexpressed and relative control T24 cells by using TRIzol reagent (Invitrogen, USA). Further transcriptome sequencing was carried out by Allwegene Tech (Beijing, China).

### Nucleic and cytoplasmic RNA

Nuclear and cytoplasmic RNA were isolated separately using the Cytoplasmic & Nucleic RNA purification kit (Norgen Biotek, Thorold, ON, USA). T24 cells were lysed in Lysis Buffer J on ice and subsequently centrifuged at 14,000 g for 10 min at 4 ℃, and transfer the supernatant containing cytoplasmic RNA to another RNase-free tube. The pellet contains the nucleic RNA. Then, the cytoplasmic and nucleic RNA were detected by qRT-PCR.

### Statistical analyses

Data were analyzed using SPSS (IBM, version 22.0). The results were recorded as mean ± standard deviation (means ± SD). Student’s t-test and one-way ANOVA were used to confirm the differences between groups. Differences were considered statistically significant at two-sided *P* values that are less than 0.05. The Kaplan–Meier method was carried out to analyse the survival condition, and log-rank test was used to analyse differences between different groups. All the data were generated from three separate experiments.

## Results

### CircFAM13B was down-regulated in BCa and positively related to lower tumour stages and prognosis in BCa patients

**Fig. 1:**
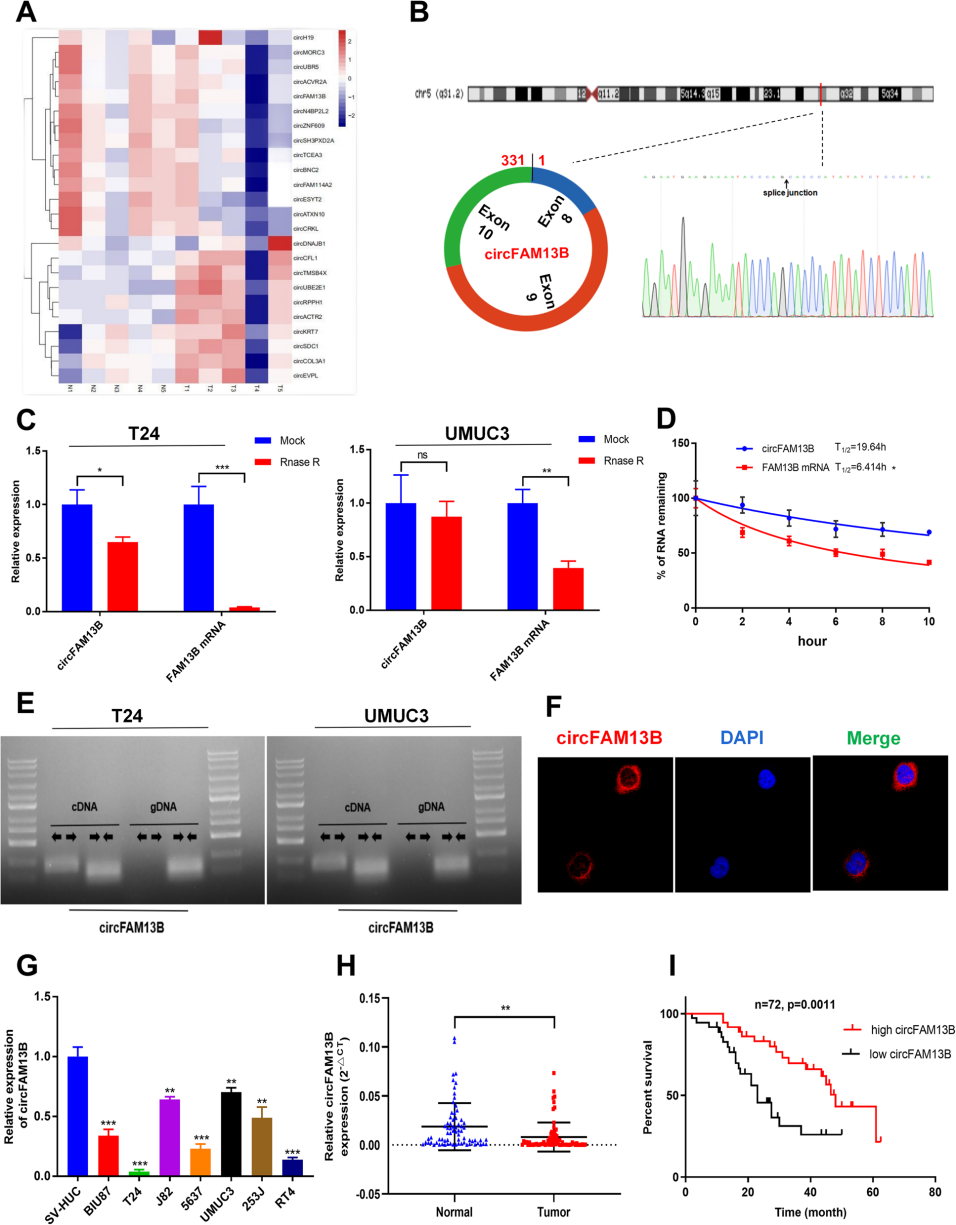
Identification and characterisation of circFAM13B in BCa. **A** Heat map of circRNA sequencing in five pairs of BCa tissues. **B** Schematic illustration that shows that circFAM13B was composed of FAM13B exon 8, exon 9 and exon 10, and the back splicing junction was confirmed by sanger sequencing. **C** The expression of circFAM13B and FAM13B mRNA in T24 and UMUC3 cells treated with or without RNase R were determined by qRT-PCR (*P < 0.05, **P < 0.01, ***P < 0.001, Student’s t-test). **D** The remaining RNA levels of circFAM13B and FAM13B mRNA in T24 cells treated with actinomycin D at different time points were determined by qRT-PCR (*P < 0.05, Student’s t-test). **E** The expression of circFAM13B in cDNA and gDNA of T24 cells were confirmed by qRT-PCR with the divergent and convergent primers. **F** The subcellular location of circFAM13B was validated by FISH experiment. **G** The expression of circFAM13B in seven BCa cell lines and SV-HUC cell line were investigated by qRT-PCR (**P < 0.01, ***P < 0.001, Student’s t-test). **H** The expression of circFAM13B in 72 pairs of BCa tissues was confirmed by qRT-PCR (**P < 0.01, Student’s t-test). **I** The relationship of circFAM13B and the overall survival of patients with BCa was confirmed by Kaplan–Meier analysis. Data are expressed as mean ± SD, *n* = 3

CircRNA sequencing was carried out on five pairs of Bca tissues and adjacent normal tissues, and differentially expressed circRNAs were screened out at fold change > 2 and *p* value < 0.05. In total, 42 significantly down-regulated circRNAs and 14 significantly up-regulated circRNAs were sorted out (Fig. 1A). Among the 10 circRNAs with the most significant down-regulation, circFAM13B had the highest average read count (> 150/tissue) in the five pairs of BCa tissues. We confirmed the circular form of circFAM13B by qRT-PCR amplification and Sanger sequencing (Fig. 1B). Rnase R and actinomycin D treatment assays were performed to validate that circFAM13B had higher stability than liner FAM13B mRNA (Fig. 1C–D). The head-to-tail splicing characteristic of circFAM13B was confirmed by qRT-PCR with divergent or convergent primers (Fig. 1E). Further FISH assays indicated that circFAM13B was mainly located in the cytoplasm (Fig. 1F). In addition, the expression of circFAM13B was confirmed down-regulated in seven BCa cell lines compared with SV-HUC (Fig. 1G). We further validated the expression of circFAM13B in 72 pairs of BCa tissues, and the results showed that circFAM13B was down-regulated in BCa tissues compared with adjacent normal tissues (Fig. 1H). The expression level of circFAM13B was negatively correlated with tumour stage in patients with BCa (Table 1). In addition, Kaplan–Meier analysis revealed that patients with higher circFAM13B expression had a better overall survival (Fig. 1I).

**Table 1:** Correlations between the expression of circFAM13B and clinicopathological features in BCa patients

Characteristics	Case	CircFAM13B		*P* value
		Low	High	
All cases	72	54	18	
Age(years)				0.6802
< 65	31	24	7	
≥ 65	41	30	11	
Gender				0.3755
Male	50	36	14	
Female	22	18	4	
Tumor stage				0.0131^*^
pTa-pT1	23	13	10	
pT2-pT4	49	41	8	
Histological grade				0.2482
Low	24	16	8	
High	48	38	10	
Tumor size(cm)				0.6681
< 3	25	18	7	
≥ 3	47	36	11	
Lymph metastasis				0.5766
No	44	32	12	
Yes	28	22	6	

^*^P < 0.05

### CircFAM13B promoted the proliferation of BCa cells

The role of circFAM13B in BCa was investigated by transfecting circFAM13B overexpression or knockdown lentivirus in T24 and UMUC3 cells without affecting the expression of FAM13B mRNA (Additional figure S1A–D). The results of CCK8 assays showed that circFAM13B inhibited the proliferation rates of T24 and UMUC3 cells (Additional figures S1E–F). The results of cloning formation assays also revealed that circFAM13B attenuated the cloning numbers of T24 and UMUC3 cells (Additional figures S1G–J).

### CircFAM13B promoted the effect of CD8^+^ T cells and immunotherapy sensitivity of BCa in vitro

**Fig. 2:**
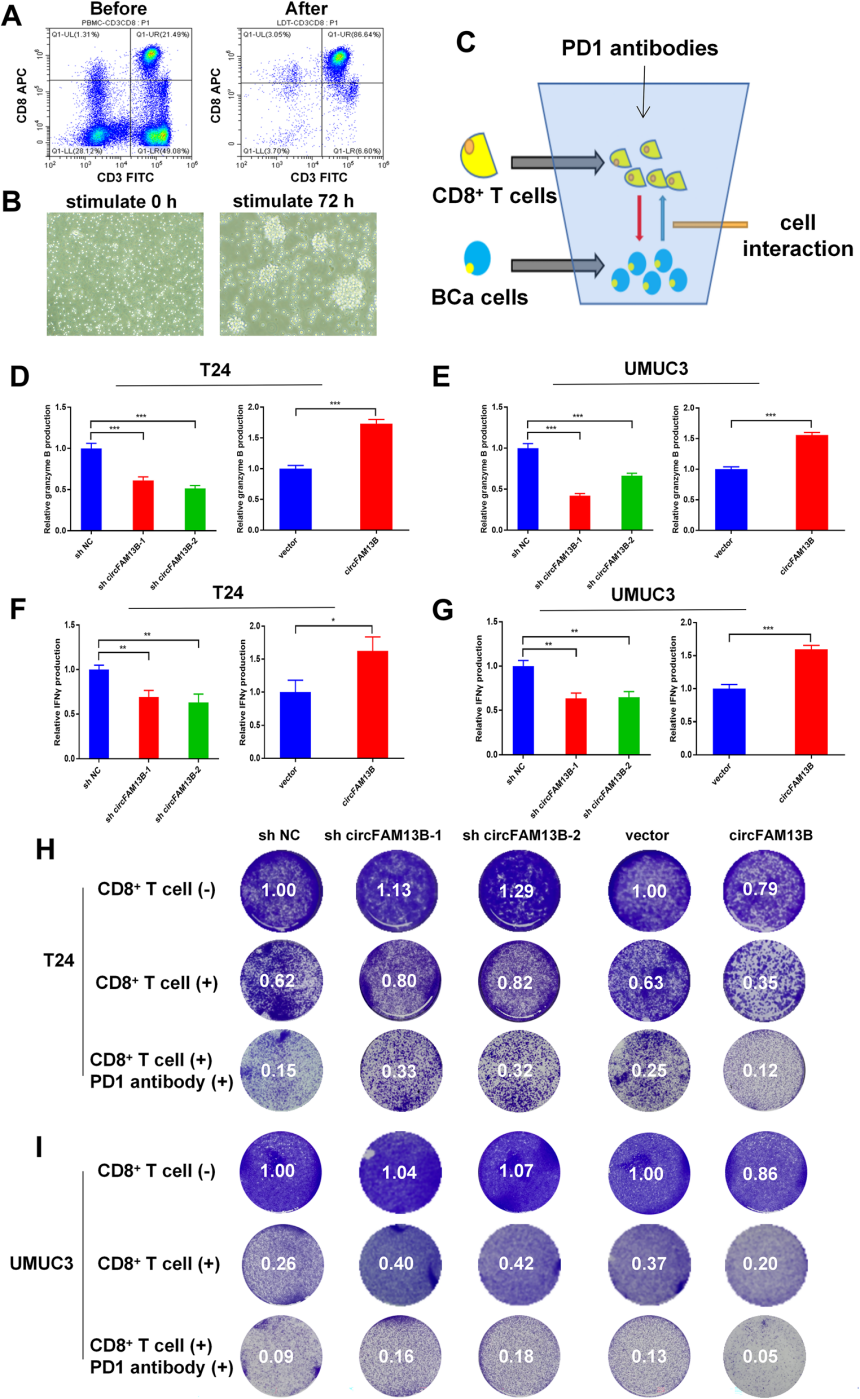
CircFAM13B promoted the effect of CD8^+^ T cells in vitro. **A** The efficiency of CD8 + cell screening was validated by flow cytometry by using CD3 and CD8 antibodies. **B** The morphology of CD8 + T cells before and after activation was observed under a microscope. **C** Schematic diagram of the co-culture model. **D-E** ELISA assays showed that CD8 + T cells co-cultured with circFAM13B knockdown T24 or UMUC3 cells secreted less granzyme B. CD8 + T cells co-cultured with circFAM13B overexpression T24 or UMUC3 cells secreted more granzyme B (***P < 0.001, Student’s t-test). **F–G** ELISA assays showed that CD8 + T cells co-cultured with circFAM13B knockdown T24 or UMUC3 cells secreted less IFN-γ. CD8 + T cells co-cultured with circFAM13B overexpression T24 or UMUC3 cells secreted more IFN-γ (*P < 0.05, **P < 0.01, ***P < 0.001, Student’s t-test). **H** The killing ability of CD8^+^ T cells and the immunotherapy sensitivity of BCa were inhibited when co-cultured with circFAM13B knockdown T24 cells. The killing ability of CD8^+^ T cells and the immunotherapy sensitivity of BCa were increased when co-cultured with circFAM13B overexpressed T24 cells. **I** The killing ability of CD8^+^ T cells and the immunotherapy sensitivity of BCa were inhibited when co-cultured with circFAM13B knockdown UMUC3 cells. The killing ability of CD8.^+^ T cells and the immunotherapy sensitivity of BCa were increased when co-cultured with circFAM13B overexpressed UMUC3 cells. Data are expressed as mean ± SD, *n* = 3

**Fig. 3:**
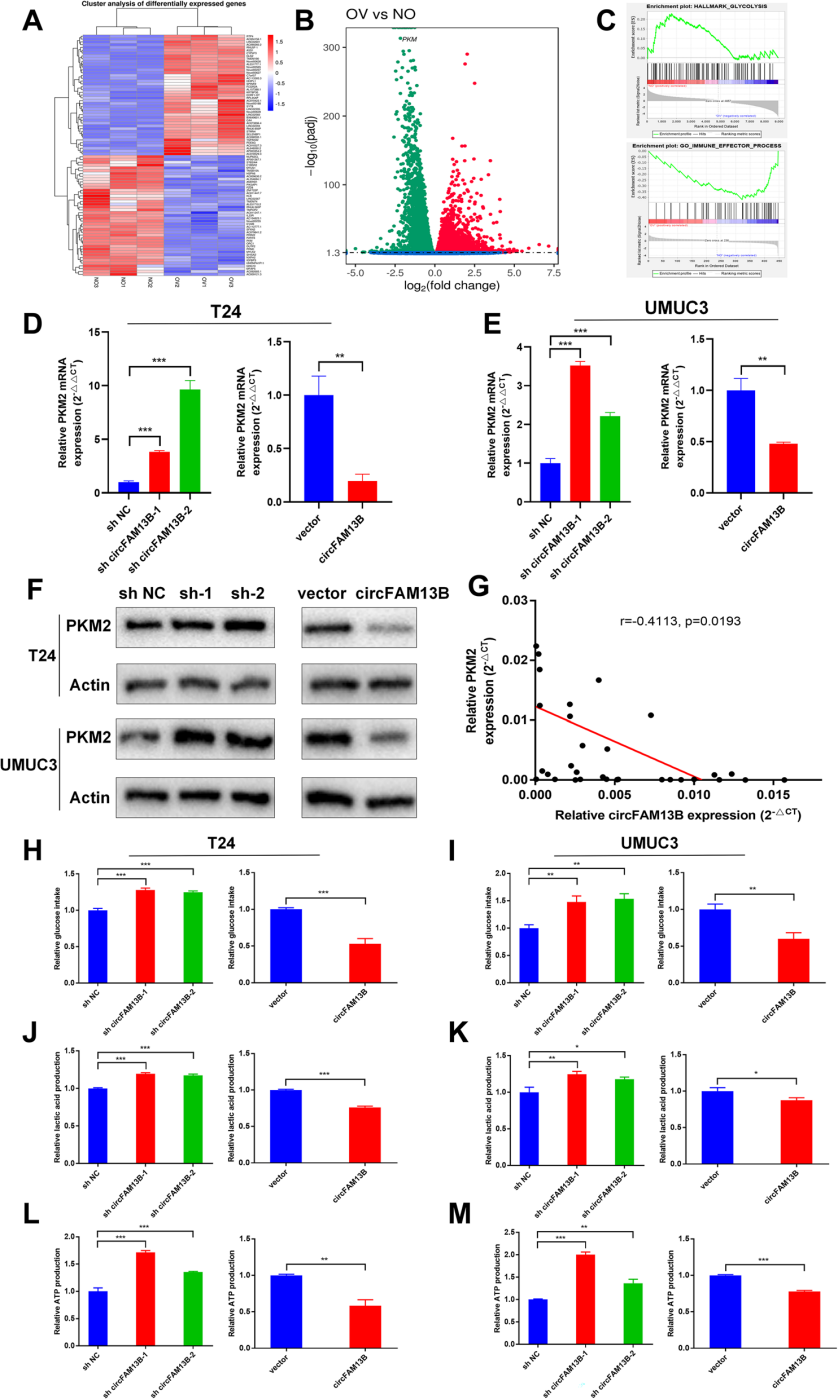
CircFAM13B inhibited the glycolysis of BCa cells by attenuating PKM2 expression. **A** Heat map of mRNA sequencing in three pairs of circFAM13B overexpression and relative control T24 cells. **B** Volcano plot of mRNA sequencing that indicates the differentially expressed genes, including PKM2. **C** GSEA analysis showed that differentially expressed genes were enriched in the glycolysis and immune response pathway. **D-E** QRT-PCR results indicated that circFAM13B inhibited the mRNA level of PKM2 in T24 and UMUC3 cells (**P < 0.01, ***P < 0.001, Student’s t-test). **F** Western blot results confirmed that circFAM13B inhibited the protein level of PKM2 in T24 and UMUC3 cells. **G.** Pearson’s correlation analysis was conducted to validate the correlation of circFAM13B and PKM2. **H-I** Glucose detection assays indicated that circFAM13B inhibited the glucose intake of T24 and UMUC3 cells (**P < 0.01, ***P < 0.001, Student’s t-test). **J-K** Lactic acid detection assays confirmed that circFAM13B inhibited the lactic acid production of T24 and UMUC3 cells (*P < 0.05, **P < 0.01, ***P < 0.001, Student’s t-test). **L-M** ATP detection assays indicated that circFAM13B inhibited the ATP production of T24 and UMUC3 cells (**P < 0.01, ***P < 0.001, Student’s t-test). Data are expressed as mean ± SD, *n* = 3

CD8^+^ T cells were sorted out from PBMCs by using CD8 microbeads (Miltenyi, Germany). The screening efficiency of CD8^+^ T cell was confirmed by flow cytometry by using CD3 and CD8 antibodies (FACs, Nanjing, China). The results showed that more than 85% of the screened cells were double positive for CD3 and CD8, which met the conditions of activation and co-culture (Fig. 2A). After activating the CD8^+^ T cells for 72 h, they showed obvious expansion and cluster growth (Fig. 2B). To determine the effect of circFAM13B on the function of CD8^+^ T cells and immunotherapy sensitivity of BCa, we co-cultured the activated CD8^+^ T cells with T24 or UMUC3 cells (Fig. 2C). The results of ELISA assay showed that CD8^+^ T cells produced less granzyme B and IFN-γ upon co-culturing with circFAM13B knockdown T24 or UMUC3 cells (Fig. 3D-G). The CD8^+^ T cells produced more granzyme B and IFN-γ upon co-culturing with circFAM13B overexpression T24 or UMUC3 cells (Fig. 2D–G). After co-culturing circFAM13B knockdown T24 or UMUC3 cells with CD8^+^ T cells for 72 h, the anti-tumour ability of CD8^+^ T cells and immunotherapy sensitivity of BCa were significantly inhibited (Figs. 2H–I). The anti-tumour ability of CD8^+^ T cells and immunotherapy sensitivity of BCa were significantly promoted when CD8^+^ T cells were co-cultured with circFAM13B overexpression T24 or UMUC3 cells (Figs. 2H–I). Subsequently, we investigated whether circFAM13B could influence the expression level of PD-L1 in T24 or UMUC3 cells. According to the western blot results, the expression of PD-L1 was unaffected by circFAM13B knockdown (Additional figure S2C-D).

### CircFAM13B inhibited the glycolytic pathway by attenuating PKM2 expression

MRNA sequencing was performed on three pairs of circFAM13B overexpression and relative control T24 cells. The differentially expressed genes were screened out, and further gene set enrichment analysis (GSEA) was conducted. In total, 1,356 genes were up-regulated, and 348 genes (including PKM2) were down-regulated in circFAM13B overexpression cells when compared with control cells (Fig. 3A–B). The GSEA results showed that circFAM13B was negatively correlated with glycolysis and positively associated with immune effect process (Fig. 3C). Gene ontology (GO) analysis and Kyoto Encyclopaedia of Genes and Genomes (KEGG) analysis were performed, and the results showed that the differentially expressed genes were enriched in metabolic process and mRNA surveillance pathways (Additional figure S2A-B). Therefore, we speculated that circFAM13B might promote immune effects by inhibiting the glycolysis-related gene, PKM2. the influence of circFAM13B to PKM2 was confirmed via qRT-PCR and Western blot assays. The results of qRT-PCR showed that circFAM13B could inhibit the expression of PKM2 mRNA (Fig. 3D–E). The results of Western blot analysis indicated that circFAM13B could attenuate the expression of PKM2 protein (Fig. 3F). The expression of PKM2 in BCa tissues was negatively associated with circFAM13B, as confirmed by Pearson’s correlation analysis in 32 samples (Fig. 3G). To confirm whether circFAM13B can affect glycolysis in BCa, we validated glucose consumption, lactic acid production and intracellular ATP production in both circFAM13B knockdown and overexpression BCa cells. The results showed that circFAM13B knockdown could promote the glucose consumption, lactic acid production and intracellular ATP production of BCa cells and vice versa (Fig. 3H–M).

The effect of PKM2 on the function of CD8^+^ T cells was determined by co-culturing the activated CD8^+^ T cells with PKM2 knockdown T24 or UMUC3 cells. The knockdown efficiency of PKM2 siRNA transfection in T24 and UMUC3 cells were confirmed by qRT-PCR (Additional figure S3A–B). The results of ELISA assays showed that CD8^+^ T cells produced more granzyme B and IFN-γ when co-cultured with PKM2 knockdown T24 or UMUC3 cells (Additional figure S3C–D). After co-culturing PKM2 knockdown T24 or UMUC3 cells with CD8^+^ T cells for 72 h, the anti-tumour ability of CD8^+^ T cells and immunotherapy sensitivity of BCa were significantly promoted (Additional figure S3E–F).

### CircFAM13B could interact with IGF2BP1 via the K homology 3–4 (KH3-4) domains

**Fig. 4:**
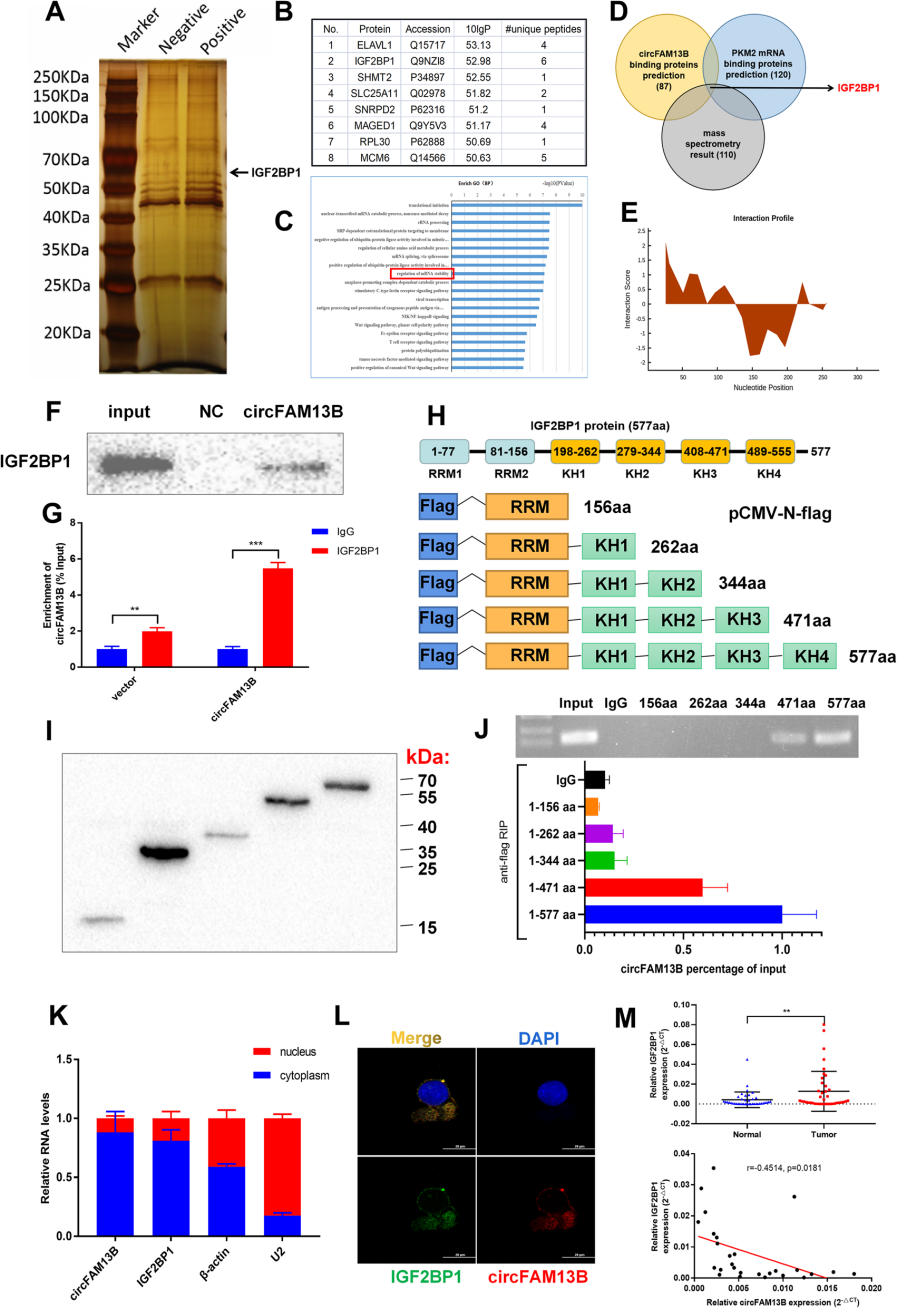
CircFAM13B interacts with IGF2BP1 protein via the KH3–4 domain. **A** RNA–protein pulldown experiment and silver staining assay were conducted to investigate the possible proteins, which could bind circFAM13B. **B** Mass spectrometry assay indicated that IGF2BP1 was pulled down by the circFAM13B probe. **C** GO enrichment analysis showed that the proteins pulled down by circFAM13B probe were enriched in the mRNA stability regulation pathway. **D** The results of StarBase database predictions and mass spectrometry analysis were intersected, and IGF2BP1 binding to both circFAM13B and PKM2 was found. **E** The binding site of circFAM13B and IGF2BP1 was predicted using the catPAPID algorithm. **F** RNA–protein pulldown and Western blot assays were performed to confirm that circFAM13B could bind to IGF2BP1. **G** RIP assays in circFAM13B overexpression and relative control T24 cells were conducted to validate the binding of IGF2BP1 and circFAM13B (**P < 0.01, ***P < 0.001, Student’s t-test). **H** Full length or truncations of flag-tagged recombinant IGF2BP1 protein vectors were designed and constructed. **I** The vectors containing full length or truncations of flag-tagged recombinant IGF2BP1 were transfected into T24 cells successfully. **J** RIP assays and qRT-PCR were carried out by using flag antibodies to determine the region of IGF2BP1 that could bind to circFAM13B. **K** Nuclear-cytoplasmic fractionation and qRT-PCR were performed to validate whether circFAM13B and IGF2BP1 are located in the nucleus or cytoplasm. **L** IF-FISH assays were carried out to indicate the co-localisation of circFAM13B and IGF2BP1 in T24 cells. **M** The expression of IGF2BP1 in BCa tissues and adjacent normal tissues were detected by qRT-PCR (**P < 0.01, Student’s t-test). Pearson’s correlation analysis was conducted to validate the correlation of circFAM13B and IGF2BP1. Data are expressed as mean ± SD, *n* = 3

CircRNAs promote the expression of downstream target genes through the miRNA sponging mechanism. However, circFAM13B inhibited the expression of PKM2, which is contrary to the miRNA-mediated sponging mechanism. Thus, circFAM13B may inhibit PKM2 expression by binding to RBP, which participates in regulating the expression of PKM2. RNA–protein pull down assay by using biotinylated probes that target the back-splicing sequence of circFAM13B was performed to verify the proteins that could bind to circFAM13B. After silver staining and mass spectrometry analysis, we screened out proteins, including IGF2BP1, that could specifically bind to circFAM13B (Fig. 4A–B). Subsequently, gene ontology (GO) analysis was performed, and the results show that proteins that could specifically bind circFAM13B were enriched in the pathway of maintaining mRNA stability (Fig. 4C). We predicted RBPs that could bind both circFAM13B and PKM2 based on the StarBase database (https://starbase.sysu.edu.cn/index.php). Then, we combined the prediction results with mass spectrometry analysis and confirmed that IGF2BP1 is an important mediator of circFAM13B in regulating PKM2 expression (Fig. 4D). We further confirmed the binding sites of IGF2BP1 on circFAM13B based on the catRAPID website (http://www.tartaglialab.com/; Fig. 4E). RNA–protein pull down and Western blot analysis were conducted to confirm that circFAM13B could bind to IGF2BP1 (Fig. 4F). RIP assay was also performed to verify that IGF2BP1 could bind to circFAM13B through by using the IGF2BP1 antibody (Fig. 4G). To confirm the specific region of IGF2BP1 that circFAM13B was bound to, we constructed plasmids containing different IGF2BP1 fragments with flag tags (Fig. 4H). After the plasmid was successfully transfected, flag antibody-mediated RIP experiment was conducted, and the results show that circFAM13B was mainly bound to the KH3 and KH4 regions of IGF2BP1 (Fig. 4I–J). The results of qRT-PCR and IF-FISH assays showed that circFAM13B and IGF2BP1 were mainly co-located in the cytoplasm (Fig. 4K–L). The expression of IGF2BP1 was detected in 40 pairs of BCa tissues, and the results showed that IGF2BP1 was highly expressed in BCa tissues compared with adjacent normal tissues (Fig. 4M). The expression of IGF2BP1 was negatively associated with circFAM13B, as confirmed by Pearson’s correlation analysis (Fig. 4M).

### CircFAM13B competitively blocked the recognition and binding of IGF2BP1 to PKM2 3’ UTR and inhibited the stability of PKM2 mRNA

**Fig. 5:**
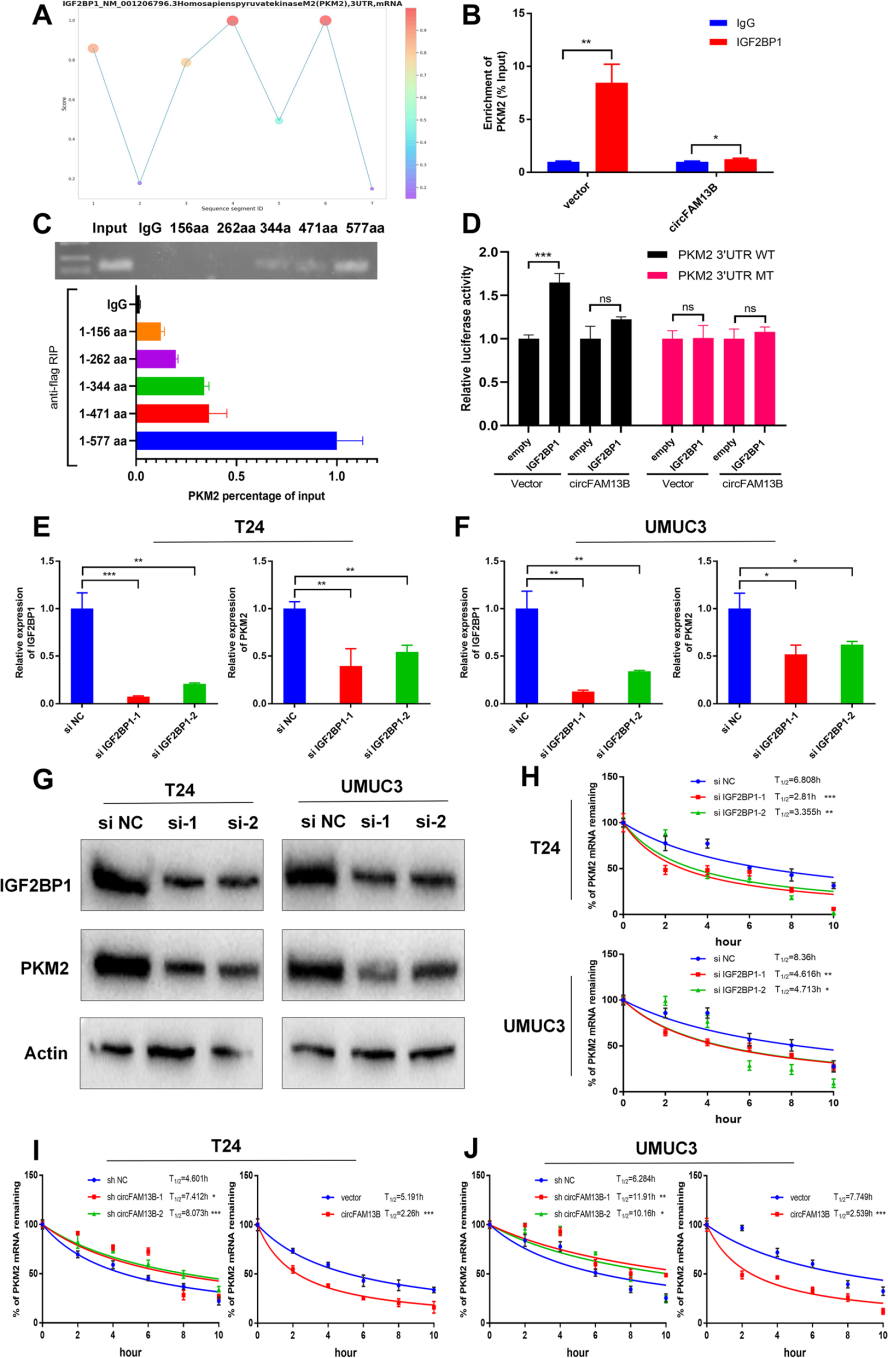
CircFAM13B inhibited the stability of PKM2 by attenuating the binding of IGF2BP1 to PKM2. **A** The binding sites of IGF2BP1 in the 3’ UTR region of PKM2 were predicted using RBPsuite. **B** RIP and qRT-PCR assays were performed to indicate the binding of IGF2BP1 protein and PKM2 3’ UTR in circFAM13B overexpression and relative control T24 cells (*P < 0.05, **P < 0.01, Student’s t-test). **C** RIP assays and qRT-PCR were carried out using flag antibodies to determine the region of IGF2BP1 that could bind to PKM2. **D** Relative luciferase activity of PKM2 3’ UTR-WT or PKM2 3’ UTR-MT that was detected in IGF2BP1 overexpression or relative control 293 T cells with or without ectopic expression of circFAM13B (***P < 0.001, Student’s t-test). **E–F** QRT-PCR assays were performed to validate the efficiency of IGF2BP1 siRNAs transfection and expression level of PKM2 in T24 and UMUC3 cells (*P < 0.05, **P < 0.01, ***P < 0.001, Student’s t-test). **G** Western blot analysis was performed to validate the efficiency of IGF2BP1 siRNAs transfection and expression level of PKM2 in T24 and UMUC3 cells. **H** The remaining PKM2 mRNA levels in IGF2BP1 siRNAs transfected or control T24 and UMUC3 cells treated with actinomycin D at different time points were determined by qRT-PCR (*P < 0.05, **P < 0.01, ***P < 0.001, Student’s t-test). **I-J** The remaining PKM2 mRNA levels in circFAM13B knockdown or overexpression T24 and UMUC3 cells treated with actinomycin D at different time points were determined by qRT-PCR (*P < 0.05, **P < 0.01, ***P < 0.001, Student’s t-test). Data are expressed as mean ± SD, *n* = 3

IGF2BP1 was bound to the 3’UTR region of PKM2 as determined using RBPsuite (http://www.csbio.sjtu.edu.cn/bioinf/RBPsuite/) (Fig. 5A). The results of RIP assays showed that IGF2BP1 could bind PKM2, but this phenomenon was inhibited when circFAM13B was overexpressed (Fig. 5B). The results of flag antibody mediated RIP confirmed that PKM2 was mainly bound to the KH4 region of IGF2BP1, which overlaps with circFAM13B to a certain extent (Fig. 5C). The results of luciferase reporter assay showed that IGF2BP1 increased the luciferase activity of PKM2 3'UTR WT plasmid, while circFAM13B overexpression inhibited this phenomenon (Fig. 5D). The small interfering RNAs (siRNAs) of IGF2BP1 were constructed and transfected into T24 and UMUC3 cells. QRT-PCR and Western blot analysis results confirmed that the expression of IGF2BP1 was inhibited by siRNA transfection, and the expression of PKM2 was attenuated because of IGF2BP1 inhibition (Fig. 5E–G). Considering that IGF2BP1 plays an important role in maintaining the stability of mRNA through KH regions, we verified the effect of IGF2BP1 on the stability of PKM2 mRNA through actinomycin treatment assays. IGF2BP1 promoted the mRNA stability of PKM2 in T24 and UMUC3 cells (Fig. 5H). Moreover, circFAM13B knockdown increased PKM2 mRNA stability and vice versa (Figs. 5I–J).

### IGF2BP1 could promote the proliferation, glycolysis and immune escape of BCa cells

**Fig. 6:**
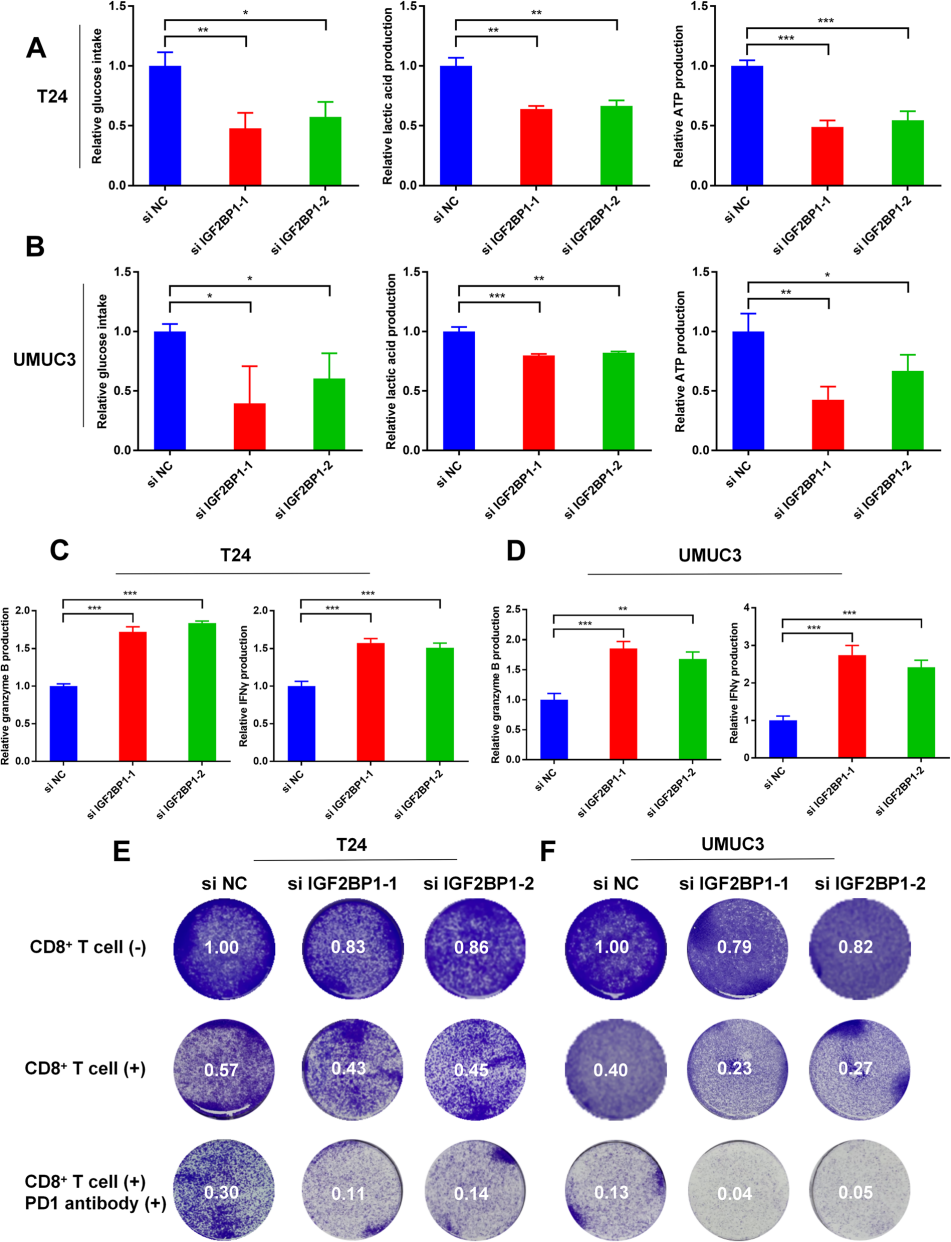
IGF2BP1 promoted the glycolysis and immune escape of BCa cells. **A-B** Glucose, lactic acid and ATP detection assays were performed in IGF2BP1 siRNAs transfected or control T24 and UMUC3 cells (*P < 0.05, **P < 0.01, ***P < 0.001, Student’s t-test). **C–D** ELISA assays were carried out to detect the granzyme B and IFN-γ produced by CD8^+^ T cells, which were co-cultured with IGF2BP1 siRNA transfected or control T24 and UMUC3 cells (**P < 0.01, ***P < 0.001, Student’s t-test). **E–F** The killing ability of CD8.^+^ T cells and the immunotherapy sensitivity of BCa were increased when co-cultured with IGF2BP1 knockdown T24 or UMUC3 cells. Data are expressed as mean ± SD, *n* = 3

The results of CCK8 assays and cloning formation assays showed that IGF2BP1 knockdown inhibited the proliferation rates of T24 and UMUC3 cells (Additional figure S4). To investigate whether IGF2BP1 can affect glycolysis in BCa, we validated glucose consumption, lactic acid production and intracellular ATP production in IGF2BP1 knockdown T24 and UMUC3 cells. The results showed that IGF2BP1 knockdown could inhibit the glucose consumption, lactic acid production and intracellular ATP production of BCa cells (Fig. 6A–B). To determine whether IGF2BP1 could affect the function of CD8^+^ T cells, activated CD8^+^ T cells were co-cultured with IGF2BP1 knockdown T24 or UMUC3 cells. The results of ELISA assays showed that CD8^+^ T cells produced more granzyme B and IFN-γ when co-culturing with IGF2BP1 knockdown T24 or UMUC3 cells (Fig. 6C–D). After co-culturing IGF2BP1 knockdown T24 or UMUC3 cells with CD8^+^ T cells for 72 h, the killing ability of CD8^+^ T cells and immunotherapy sensitivity of BCa were substantially promoted (Fig. 6E–F).

### IGF2BP1 overexpression rescued the repressed proliferation, glycolysis and immune escape induced by circFAM13B in BCa

**Fig. 7:**
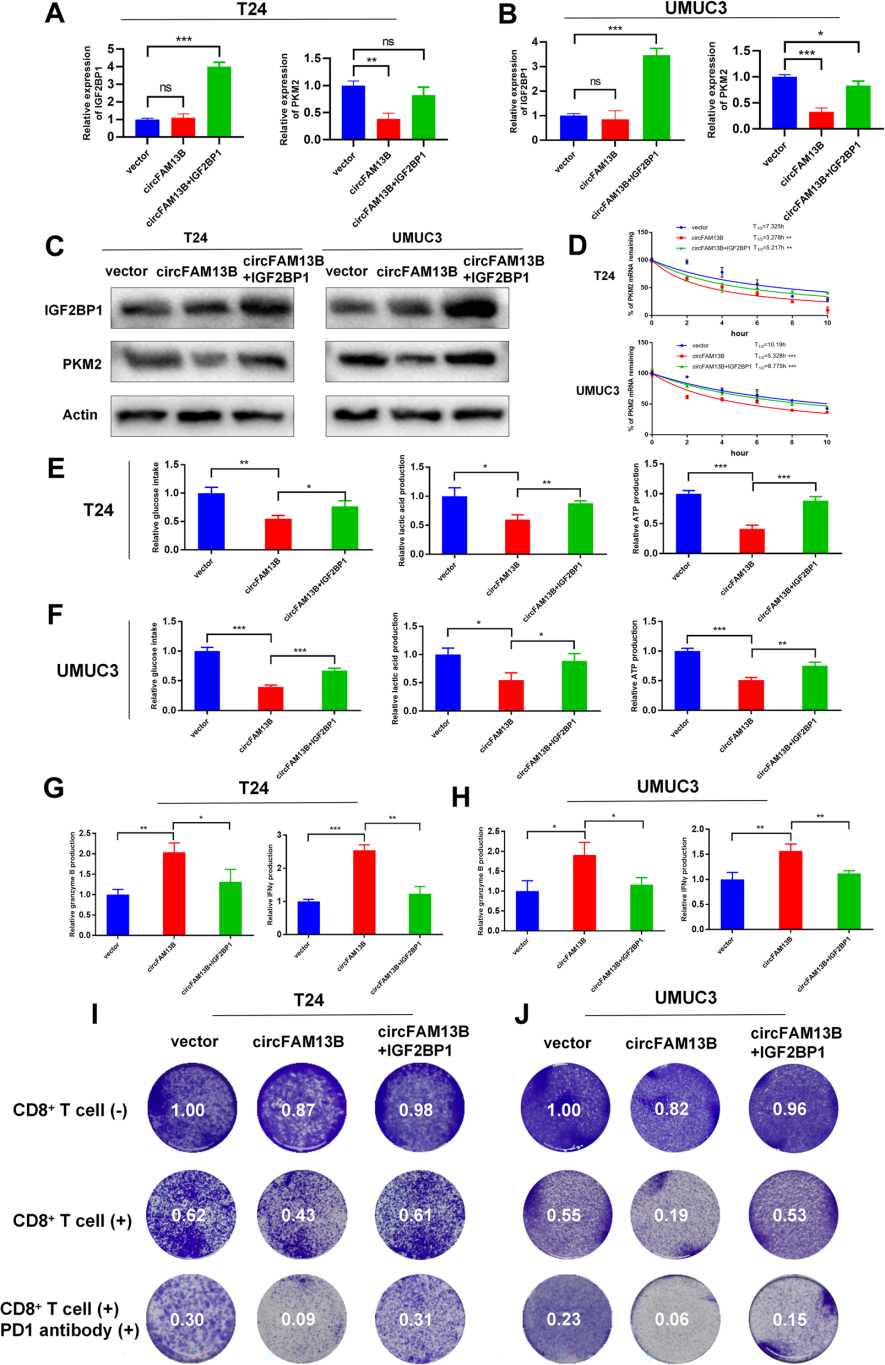
IGF2BP1 rescued the decreased PKM2 stability, repressed glycolysis and inhibited immune escape induced by circFAM13B. **A-B** QRT-PCR assays showed that the overexpression of IGF2BP1 rescued the attenuation of PKM2 mRNA expression caused by circFAM13B in T24 and UMUC3 cells. (*P < 0.05, **P < 0.01, ***P < 0.001, Student’s t-test). **C** Western blot assays showed that the overexpression of IGF2BP1 rescued the attenuation of PKM2 protein expression caused by circFAM13B in T24 and UMUC3 cells. **D** Actinomycin D treatment assays showed that the overexpression of IGF2BP1 rescued the inhibition of mRNA stability caused by circFAM13B in T24 and UMUC3 cells (**P < 0.01, ***P < 0.001, Student’s t-test). **E–F** Glucose, lactic acid, and ATP detection assays showed that the overexpression of IGF2BP1 rescued the inhibition of glycolysis caused by circFAM13B in T24 and UMUC3 cells (*P < 0.05, **P < 0.01, ***P < 0.001, Student’s t-test). **G–H** ELISA assays showed that the overexpression of IGF2BP1 weakened the enhancement of granzyme B and IFN-γ production of CD8 + T cells caused by co-culturing with circFAM13B overexpressed T24 and UMUC3 cells (*P < 0.05, **P < 0.01, ***P < 0.001, Student’s t-test). **I–J** Overexpression of IGF2BP1 weakened the enhanced CD8.^+^ T cells killing ability and increased the immunotherapy sensitivity of BCa cells caused by co-culturing with circFAM13B overexpressed T24 and UMUC3 cells. Data are expressed as mean ± SD, *n* = 3

QRT-PCR and Western blot analysis results revealed that circFAM13B inhibited the expression of PKM2 without influencing the expression of IGF2BP1, while the transfection of IGF2BP1 overexpression plasmid in circFAM13B overexpression T24 and UMUC3 cells could rescue the repressed PKM2 expression (Fig. 7A–C). The results of actinomycin treatment assays showed that co-transfection of IGF2BP1 and circFAM13B in T24 and UMUC3 cells could rescue the decreased PKM2 mRNA stability induced by circFAM13B overexpression (Fig. 7D). CCK8 assays and cloning formation assays confirmed that the co-transfection of IGF2BP1 and circFAM13B in T24 and UMUC3 cells could rescue the inhibited proliferation rates induced by circFAM13B overexpression (Additional figure S5). The inhibited glucose consumption, lactic acid production and intracellular ATP production in circFAM13B overexpression T24 and UMUC3 cells were rescued by IGF2BP1 transfection (Fig. 7E–F).

The role of circFAM13B-IGF2BP1 pathway in BCa immune escape and immunotherapy sensitivity was determined by co-transfecting circFAM13B and IGF2BP1 in T24 and UMUC3 cells and co-culturing them with activated CD8^+^ T cells. The results of ELISA assays showed that IGF2BP1 weakened the increased granzyme B and IFN-γ production induced by circFAM13B (Fig. 7G–H). The elevated anti-tumour ability of CD8^+^ T cells and the increased immunotherapy sensitivity of BCa induced by circFAM13B were also weakened by IGF2BP1 transfection (Fig. 7I–J).

### CircFAM13B overexpression augmented the effect of CD8^+^ T cells and efficacy of anti-PD-1 therapy in HuNOG mice model

**Fig. 8:**
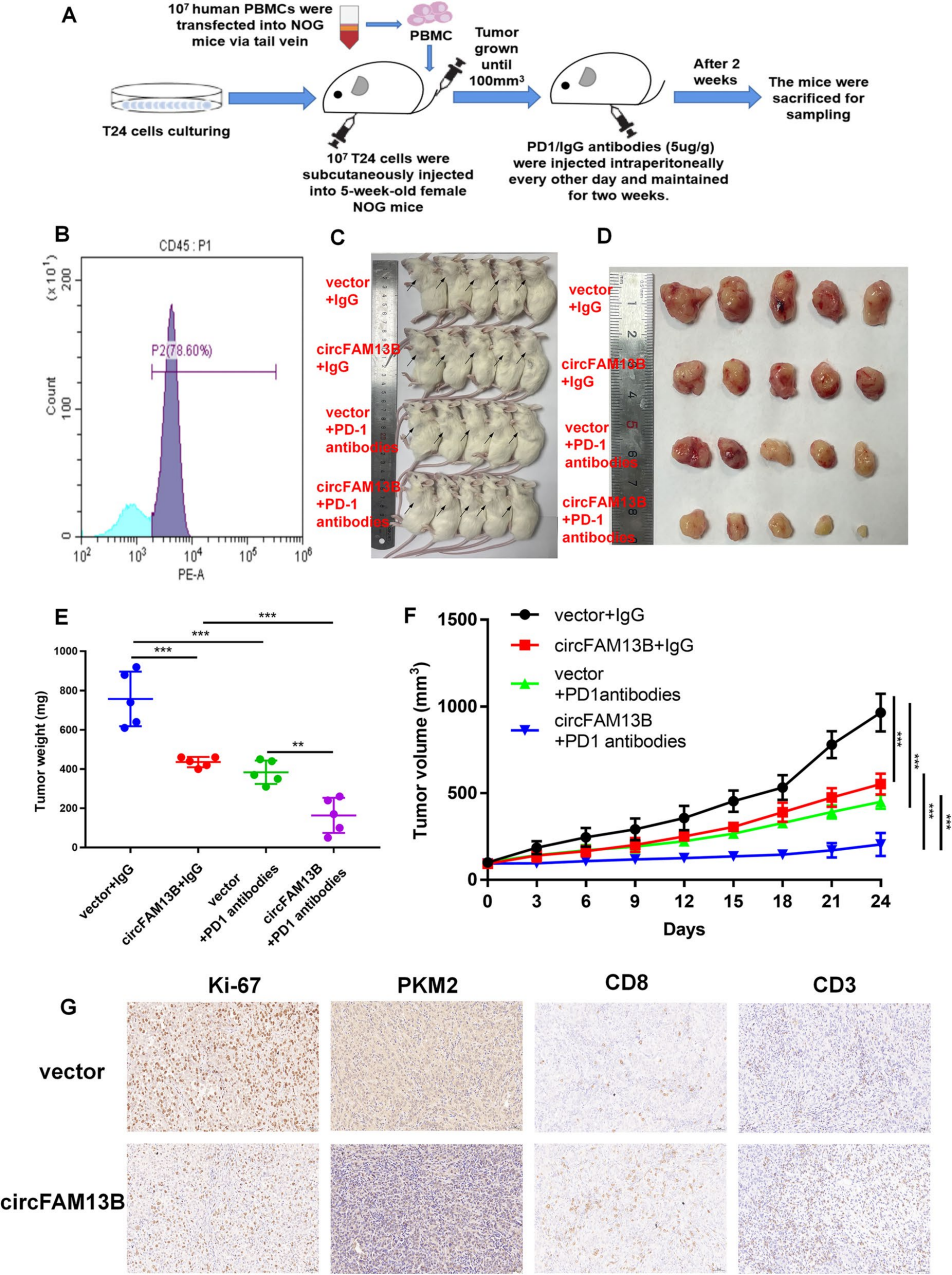
CircFAM13B overexpression augments the efficacy of anti PD-1 therapy in HuNOG mice model. **A** Schematic of the huNOG mice model establishment and experimental design to investigate the role of circFAM13B in regulating immune escape and anti-PD-1 therapy efficacy in vivo. **B** Flow cytometry was performed to detect the positive rate of human CD45 in the HuNOG mice model. **C** Representative image of HuNOG mice injected with circFAM13B or relative control T24 cells, which were treated with PD-1 or IgG antibodies (*n* = 5). **D** Representative image of the tumour formation of HuNOG mice injected with circFAM13B or relative control T24 cells, which were treated with PD-1 antibodies or (*n* = 5). **E** The weights of the tumours removed from HuNOG mice were measured using electronic scales (**P < 0.01, ***P < 0.001, Student’s t-test). **F** The tumour volumes of HuNOG mice were measured every 3 days (***P < 0.001, Student’s t-test). **G** Representative images of IHC staining of Ki-67, PKM2, CD8 and CD3 in tumour samples removed from HuNOG mice. Data are expressed as mean ± SD, *n* = 3

The role of circFAM13B in anti-tumour function of CD8^+^ T cells and sensitivity of immunotherapy was investigated in vivo by designing and constructing HuNOG mice model as previously described (Fig. 8A). To confirm the success of huNOG model construction, we detected the proportion of human CD45-positive cells in the peripheral blood of mice by flow cytometry. The result confirmed that more than 78% of the PBMCs were human CD45 positive (Fig. 8B). We divided huNOG mice inoculated with circFAM13B overexpression or control T24 cells into four groups, namely, circFAM13B + PD-1 antibody, vector + PD-1 antibody, circFAM13B + IgG and vector + IgG group. Four weeks after the initial subcutaneous injection of PD-1 antibodies, huNOG mice were sacrificed, and the tumours were dissected (Fig. 8C–D). Based on the detection of the tumour volume and quality, circFAM13B could significantly inhibit BCa growth in huNOG mice and promote the immunotherapy sensitivity of BCa (Fig. 8E–F). The results of IHC staining showed that the expression of Ki-67 and PKM2 were inhibited by circFAM13B in huNOG tumours (Fig. 8G). The IHC staining results also indicated that circFAM13B increased the positive rates of CD8 and CD3 in huNOG tumours, suggesting that more CD8^+^ T cells were infiltrated in circFAM13B overexpressed tumours (Fig. 8G). qRT-PCR and western blotting confirmed the expression of circFAM13B, IGF2BP1, and PKM2 in tumors of NOG mice. The results showed PKM2 expression was inhibited when circFAM13B was overexpressed. While the expression of IGF2BP1 was unaffected by circFAM13B in vivo (Additional figure S5 E–F).

### HNRNPL promoted the back-splicing of circFAM13B

**Fig. 9:**
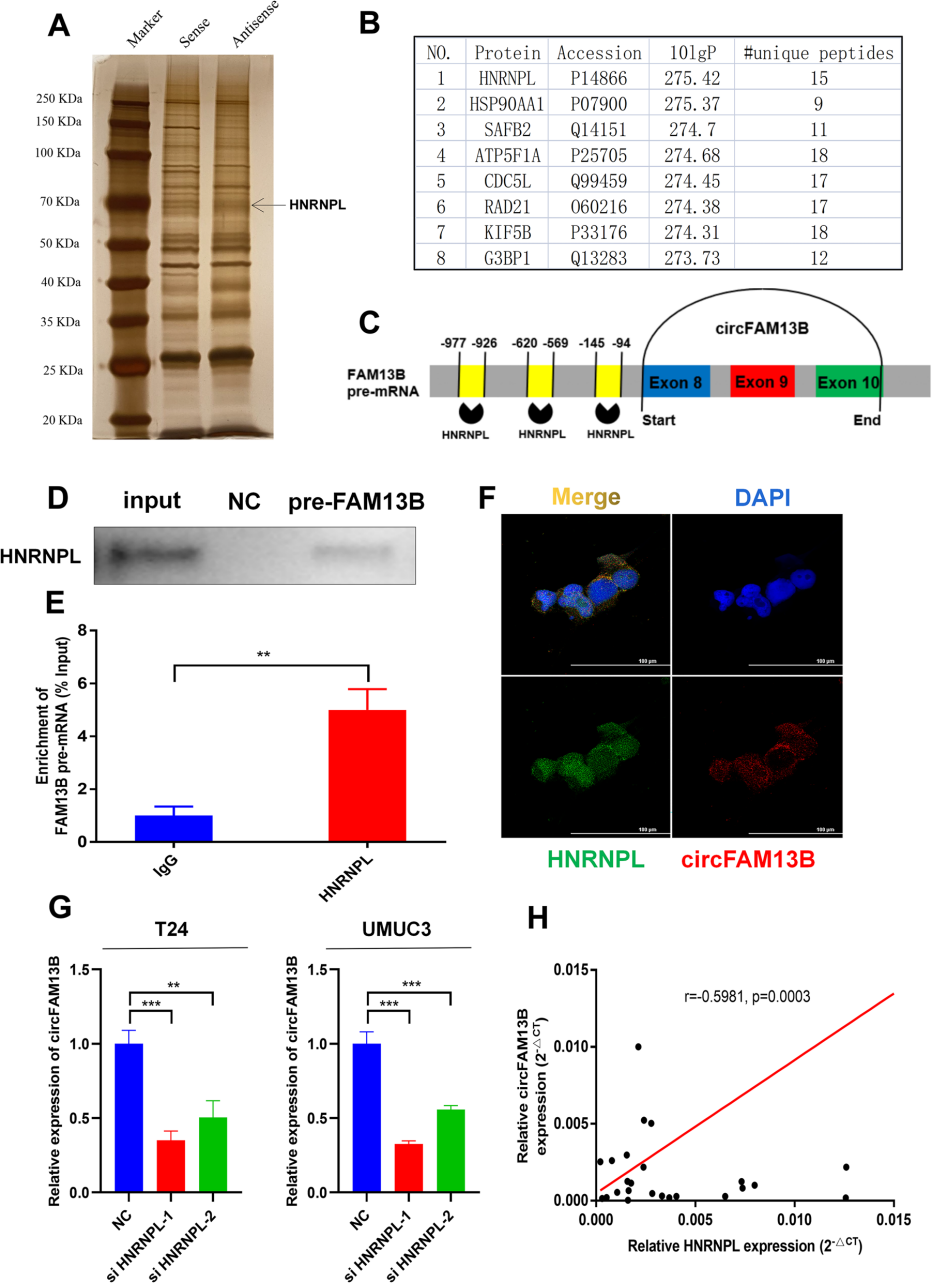
HNRNPL induced the back-splicing of circFAM13B. **A** RNA–protein pulldown experiment and silver staining assay were conducted to investigate the possible proteins, which could bind the flanking introns of circFAM13B. **B** Mass spectrometry assay results indicated that HNRNPL was pulled down by the probes of flanking introns of circFAM13B. **C** The binding sites of HNRNPL in the flanking introns sequences of the pre-FAM13B mRNA were predicted via catRAPID. **D** RNA–protein pulldown and Western blot assays were performed to confirm that the flanking introns of circFAM13B could bind to HNRNPL. **E** RIP assay in T24 cells was conducted to validate the binding of HNRNPL and flanking introns of circFAM13B (**P < 0.01, Student’s t-test). **F** IF-FISH assays were carried out to indicate the co-localisation of circFAM13B and HNRNPL in T24 cells. **G** QRT-PCR assays were performed to validate the expression level of circFAM13B in HNRNPL knockdown T24 and UMUC3 cells (**P < 0.01, ***P < 0.001, Student’s t-test). **H** Pearson’s correlation analysis was conducted to validate the correlation of circFAM13B and HNRNPL. Data are expressed as mean ± SD, *n* = 3

The mechanism in which circFAM13B was generated was determined by designing and constructing the biotin-labelled probe, which could bind the flanking intron sequence of circFAM13B. RNA–protein pull down, silver staining and mass spectrometry analysis were performed, and the results show that HNRNPL could specifically bind to the flanking introns of circFAM13B (Fig. 9A–C). Based on GO analysis, proteins that specifically bind the flanking introns of circFAM13B were enriched in the RNA splicing pathway (Additional figure S6A). The results of mass spectrometry analysis (–lg10 ≥ 20, unique peptides ≥ 10) were intersected with circFAM13B back-splicing relative RBPs predicted using the RNAct website (https://rnact.crg.eu/). The results show that ADAR1 and HNRNPL may be involved in the formation of circFAM13B (Additional figure S6B). ADAR1 was excluded, because the expression level of circFAM13B did not change after knocking down ADAR1 by siRNAs in T24 cells and UMUC3 cells (Additional figure S6C).

RNA–protein pull down and Western blot were conducted to confirm that the flanking introns of circFAM13B could bind to HNRNPL (Fig. 9D). RIP assay was performed to confirm that HNRNPL could bind to the flanking introns of circFAM13B by using the HNRNPL antibody (Fig. 9E). IF-FISH assay results showed that circFAM13B and HNRNPL were co-localised in both the nucleus and cytoplasm (Fig. 9F). The HNRNPL siRNAs were constructed and transfected into the T24 and UMUC3 cells. The knockdown efficiency of HNRNPL siRNAs were proved by qRT-PCR and Western blot analysis (Figures S6D–E). The results of qRT-PCR showed that the knockdown of HNRNPL inhibited the expression of circFAM13B in T24 and UMUC3 cells (Fig. 9G). We further confirmed that the expression of circFAM13B was positively associated with HNRNPL in BCa tissues based on Pearson’s correlation analysis (Fig. 9H).

## Discussion

In the present research, we identified circFAM13B from the circRNA sequencing of five pairs of BCa tissues. We further verified the low expression of circFAM13B in BCa tissues and cell lines collected. The results indicate that circFAM13B was correlated with improved overall survival and lower tumour stage. In vitro and in vivo studies confirmed that circFAM13B inhibited the acidic TME of BCa and promoted the infiltration and activity of CD8^+^ T cells by inhibiting the stability of PKM2 mRNA by competitively binding to IGF2BP1 KH3-4 domains. By administrating PD-1 antibodies into the co-culturing system and the humanised NOG mice inoculated with BCa tumours, we found that circFAM13B could enhance the sensitivity of immunotherapy. Therefore, a novel mechanism of circRNA-induced immune strengthening was characterised in BCa, and the results indicate that circFAM13B may act as an important biomarker to predict the susceptibility of patients with BCa to immunotherapy.

Tumour cells always exhibit abnormal metabolic status during which glycolysis is enhanced and lactic acid accumulates [30]. Lactic acid can decrease the pH value of TME and promote immune escape by inhibiting the infiltration and proliferation of immune cells, especially CD8^+^ T cells [31, 32]. Specifically, acidic TME can inhibit CD8^+^ T cell function in several ways. First, the accumulation of external lactic acid and acidic TME will affect the normal metabolic state of CD8^+^ T cells, such as TCA cycle and lactate excretion, thus downregulating CD8^+^ T cell activity [33, 34]. In addition, the production of some tumour-killing cytokines such as IFN-γ is inhibited by lactic acid and acidic TME [35, 36]. Other than CD8^+^ T cells, lactic acid can also recruit and activate immune suppressive cells, such as Treg cells, MDSCs and tumour-associated macrophages (TAMs), to further inhibit antitumor responses [37]. As reported, lactic acid in the low-glucose TME could be taken up and utilized by Treg cells, which promoted the expression of PD-1. PD-1 blockade immunotherapy could stimulate these Treg cells, thereby diminishing the treatment's efficacy [38, 39]. Therefore, factors that can affect glycolysis and acidic TME could modulate the immune escape and immunotherapy sensitivity. Many enzymes are involved in glycolysis, and PKM2 is the core enzyme that affects the last step of glycolysis, resulting in the production and accumulation of lactic acid [12]. PKM2 is highly expressed in BCa tissues and is associated with the poor prognosis of patients with BCa [17]. PKM2 can promote the proliferation of BCa cells, which has also been proved in our previous study [17]. Moreover, PKM2 can be involved in the regulation of immune response to some extent [13, 15, 40]. Therefore, PKM2-mediated glycolytic pathway and acidic TME are the key regulatory factors for immune escape and immunotherapy resistance.

An increasing number of studies supports that circRNA is an important factor that is involved in tumorigenesis and development [41]. Considering the stable structure of the circRNA, it has better potential than linear RNA as a molecular target for tumour diagnosis and treatment [42]. Recent studies suggest that circRNA is involved in the regulation of tumour immune escape and immunotherapy sensitivity [43, 44]. Previous studies focused mostly on the direct regulation of PD-L1 expression by circRNA [45–47]. For instance, circIGF2BP3, circFGFR1 and circ-CPA4 could promote immune evasion and inhibit CD8^+^ T cell response in non-small cell lung cancer by facilitating the PD-L1 expression [45–47]. Studies have not investigated how circRNA regulates immune response by affecting tumour glycolysis and acidic TME. In the present study, circFAM13B inhibited the immune escape and promoted the immunotherapy sensitivity of BCa by inhibiting PKM2-mediated glycolysis and acidic TME. circRNA often promotes the expression of downstream target genes through miRNA-mediated sponging mechanism [48], which is contrary to our verification result. The open reading frame (ORF) was not observed in the sequence of circFAM13B in the CSCD database (http://gb.whu.edu.cn/CSCD/). Therefore, circFAM13B may attenuate PKM2 expression through another important pathway in combination with a RBP, which participates in the regulation of PKM2 expression. As an RBP, IGF2BP1 plays important roles in embryogenesis, carcinogenesis and chemo-resistance by influencing the stability, translation and localisation of mRNA [49–53]. IGF2BP1 has six RNA binding domains with different functions, including two RNA-recognition motifs (RRMs) in the N-terminal and four K homology (KH) domains, which bind mRNA mostly in the C-terminal [49]. The KH domains of IGF2BP1, especially the KH3-4 domains, play essential roles that bind mRNAs in an m6A-dependent pathway [54]. In BCa, IGF2BP1 promotes tumour proliferation, migration and invasion by regulating the expression of MYC and FSCN1 [24]. IGF2BP1 facilitates the glycolysis of colon cancer by binding and stabilising the LDHA, which could promote the lactic acid accumulation and acidic TME production [25, 55, 56]. According to in vitro functional assays, IGF2BP1 could promote the glycolysis, immune escape and decrease in the immunotherapy sensitivity of BCa cells by stabilising the PKM2 mRNA. The function of IGF2BP1 can be regulated by circRNAs by attenuating the functional KH domains [24]. In the present study, circFAM13B blocked the function of IGF2BP1 by occupying the KH3-4 domains, thus decreasing the binding of IGF2BP1 to PKM2.

In addition to exploring the effect of circFAM13B on BCa immune response, we also investigated the formation of circFAM13B. We screened the RBPs that could bind to the flanking introns of circFAM13B, and this mechanism is important for circRNA generation [57, 58]. HNRNPL, as a member of HNRNP family that is involved in the alternatively splicing of pre-mRNA [27], was selected and confirmed to promote circFAM13B production. HNRNPL can recognise and bind to the flanking GC-enriched intron regions of pre-mRNA specially, resulting in the back-splicing of circRNAs [27]. In addition to circFAM13B, HNRNPL modulates the production of circANKRD42 and circARHGAP35 [28, 29]. This finding provides a direction for a better understanding of the formation of circRNAs.

The limited understanding of the TME dynamics hinders the improvement of immunotherapy sensitivity. Considering that the important regulatory role of circRNA on TME has been revealed, its influence on immunotherapy sensitivity has also emerged. With more meaningful research on circRNA and immunotherapy appearing, new effective biomarkers for the management of BCa can be discovered.

## Conclusions

**Fig. 10:**
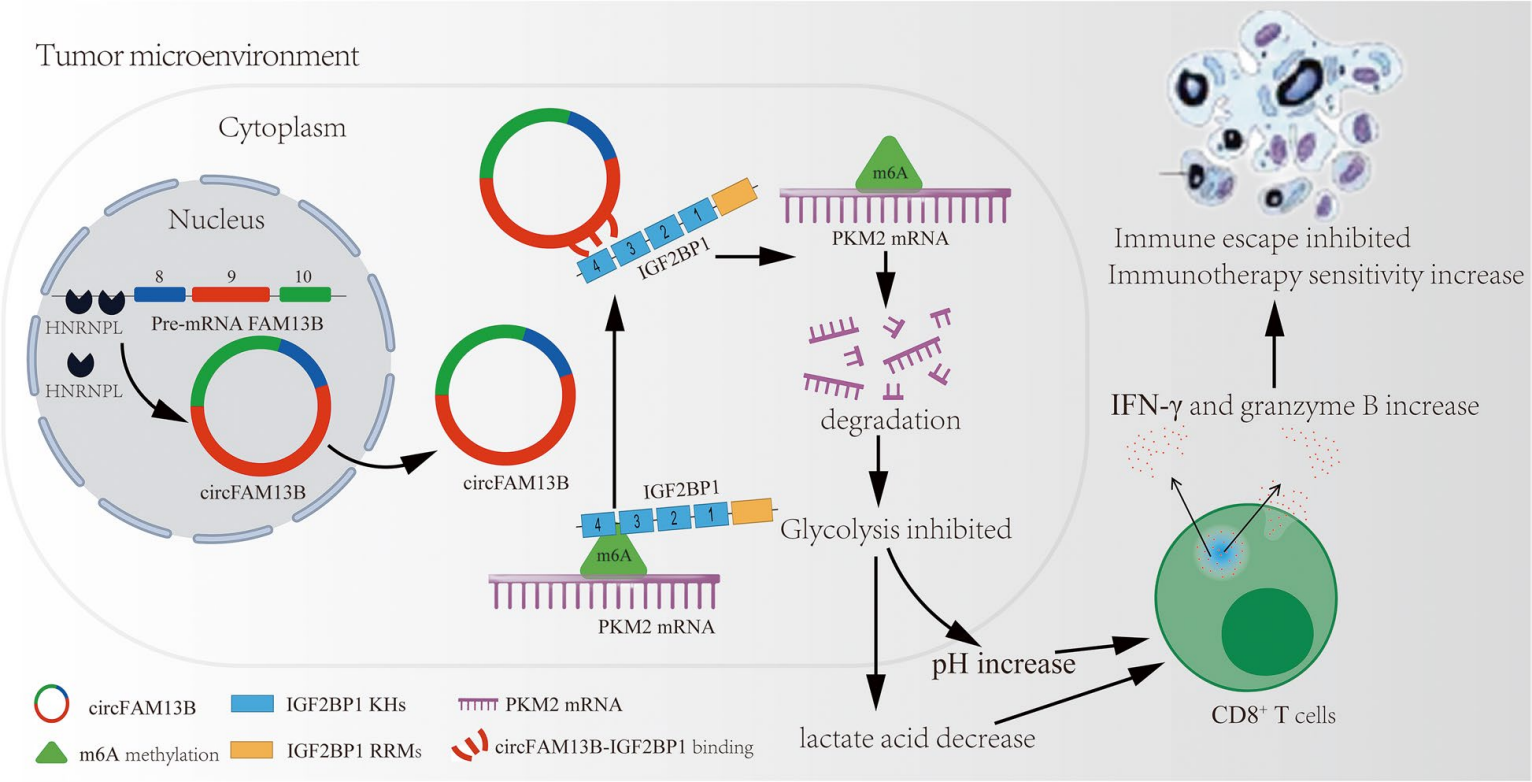
Mode pattern of the HNRNPL-induced circFAM13B/IGF2BP1/PKM2 regulatory and function network

Mechanistically, our study showed that HNRNPL-induced circFAM13B could inhibit the stability of PKM2 mRNA by competitively binding to IGF2BP1 in BCa. Functionally, circFAM13B inhibited the proliferation and immune escape and increased the immunotherapy sensitivity of BCa by attenuating glycolysis. The mechanism and function network of circFAM13B is shown in Fig. 10. In summary, circFAM13B has great potential in predicting immunotherapy sensitivity and acting as a new therapeutic target in BCa.

## Supplementary Information

**Additional file 1: Table S1.** All siRNAs/shRNAs used in this research.

**Additional file 2: Table S2.** All PCR primers used in this research.

**Additional file 3: Figure S1.** CircFAM13B inhibited the proliferation of BCa cells. A**-**B. The efficiency of circFAM13B knockdown in T24 and UMUC3 cells was verified by qRT-PCR (**P<0.01, ***P<0.001, Student’s t-test). C–D. The efficiency of circFAM13B overexpression in T24 and UMUC3 cells was verified by qRT-PCR (***P<0.001, Student’s t-test). E. CCK8 assays showed that circFAM13B inhibited the proliferation of T24 cells (***P<0.001, Student’s t-test). F. CCK8 assays showed circFAM13B inhibited the proliferation of UMUC3 cells (**P<0.01, ***P<0.001, Student’s t-test). G. Colony formation assays confirmed circFAM13B inhibited the proliferation of T24 cells. H. Colony formation assays confirmed that circFAM13B inhibited the proliferation of UMUC3 cells. I–J. Histograms of colony formation assays (*P<0.05, **P<0.01, Student’s t-test). Data are expressed as mean±SD, *n*=3.

**Additional file 4: Figure S2.** GO and KEGG analysis of circFAM13B relative mRNA sequencing. A. GO analysis was conducted on the differentially expressed genes of mRNA sequencing. B. KEGG analysis was conducted on the differentially expressed genes of mRNA sequencing. C. Western blot was conducted to investigate the influence of circFAM13B on PD-L1 in T24 cells. D. Western blot was conducted to investigate the influence of circFAM13B on PD-L1 in UMUC3 cells.

**Additional file 5: Figure S3.** PKM2 knockdown inhibited the immune escape of BCa cells. A**-**B. QRT-PCR assays were performed to validate the efficiency of PKM2 siRNAs transfection in T24 and UMUC3 cells (***P<0.001, Student’s t-test). C–D. ELISA assays were carried out to detect the granzyme B and IFN-γ produced by CD8+ T cells, which were co-cultured with PKM2 siRNAs transfected or control T24 and UMUC3 cells (*P<0.05, **P<0.01, ***P<0.001, Student’s t-test). E–F. The killing ability of CD8^+^ T cells and the immunotherapy sensitivity of BCa were increased after being co-cultured with PKM2 knockdown T24 or UMUC3 cells. Data are expressed as mean±SD, *n*=3.

**Additional file 6: Figure S4.** IGF2BP1 promoted the proliferation of BCa cells. A**-**B. CCK8 assays were carried out in IGF2BP1 siRNAs transfected or control T24 and UMUC3 cells (***P<0.001, Student’s t-test). C–D. Colony formation assays were performed in IGF2BP1 siRNAs transfected or control T24 and UMUC3 cells (**P<0.01, Student’s t-test). Data are expressed as mean±SD, *n*=3.

**Additional file 7: Figure S5.** IGF2BP1 overexpression rescued the repressed proliferation induced by circFAM13B in BCa. A**-**B. CCK8 assays showed that the overexpression of IGF2BP1 rescued the inhibition of proliferation caused by circFAM13B in T24 and UMUC3 cells (**P<0.01, ***P<0.001, Student’s t-test). C–D. Colony formation assays showed that the overexpression of IGF2BP1 rescued the inhibition of proliferation caused by circFAM13B in T24 and UMUC3 cells (*P<0.05, **P<0.01, Student’s t-test). E. qRT-PCR confirmed circFAM13B inhibited PKM2 expression in tumors of NOG mice without influencing the expression of IGF2BP1 (***P<0.001, Student’s t-test). F. Western blot confirmed circFAM13B inhibited PKM2 expression in tumors of NOG mice without influencing the expression of IGF2BP1. Data are expressed as mean±SD, *n*=3.

**Additional file 8: Figure S6.** Construction of HNRNPL knockdown T24 and UMUC3 cells. A. GO analysis was conducted on the proteins pulled down by flanking introns probe. B. The results of RNAct database predictions and circFAM13B flanking introns relative mass spectrometry analysis were intersected, and HNRNPL and ADAR1 were found. C. The expression of ADAR1 and circFAM13B in T24 or UMUC3 cells were confirmed by qRT-PCR after ADAR1 siRNA transfection (**P<0.01, ***P<0.001, Student’s t-test). D. The knockdown efficiency of HNRNPL siRNAs transfection in T24 and UMUC3 cells were confirmed by qRT-PCR (***P<0.001, Student’s t-test). E. The knockdown efficiency of HNRNPL siRNAs transfection in T24 and UMUC3 cells were confirmed by Western blot analysis. Data are expressed as mean±SD, *n*=3.

## Data Availability

The datasets supporting the conclusions of this article are included within the article and its additional files.

## Abbreviations

BCa: Bladder cancer
CircRNA: Circular RNA
TME: Tumour microenvironment
NMIBC: Non-muscle invasive bladder cancer
MIBC: Muscle invasive bladder cancer
ICIs: Immune checkpoint inhibitors
Treg: Regulatory T cells
MDSC: Myeloid-derived suppressor cells
TAMs: Tumour-associated macrophages
PKM2: Pyruvate kinase muscle isozyme M2
MiRNAs: MicroRNAs
RBPs: RNA binding proteins
IGF2BP1: Insulin-like growth factor-2 mRNA-binding protein 1
M6A: N6-methyladenosine
HNRNPL: Heterogeneous nuclear ribonucleoprotein L
qRT-PCR: Quantitative real-time PCR
RRMs: RNA-recognition motifs
KH: K homology
IF-FISH: Immunofluorescence assay and fluorescence in situ hybridization
RIP: RNA immunoprecipitation
ATP: Adenosine triphosphate
PBMCs: Peripheral blood mononuclear cells
HuNOG mice: Human induced NOD-scid IL2Rgnull mice
IHC: Immunohistochemistry
