# Supplementary material for: HNRNPL induced circFAM13B increased bladder cancer immunotherapy sensitivity via inhibiting glycolysis through IGF2BP1/PKM2 pathway
Source: J Exp Clin Cancer Res. 2023 Feb 6;42:41. doi: 10.1186/s13046-023-02614-3 (PMC9901087; doi:10.1186/s13046-023-02614-3)
Supplement: Supplementary file 1 — Additional file 1: Table S1. All siRNAs/shRNAs used in this research. [file 13046_2023_2614_MOESM1_ESM.doc]

**Additional file 1: Table S1:** All siRNAs/shRNAs used in this research.

| **Gene/circRNA** | **Sequences (5’-3’)** |
| --- | --- |
| CircFAM13B shRNA-1 top  CircFAM13B shRNA-1 bottom | GATCCGAAAATACCCAGCACCCATATATCTTTCAAGAGAAGATATATGGGTGCTGGGTATTTTCTTTTTTG  AATTCAAAAAAGAAAATACCCAGCACCCATATATCTTCTCTTGAAAGATATATGGGTGCTGGGTATTTTCG |
| CircFAM13B shRNA-2 top  CircFAM13B shRNA-2 bottom | GATCCGAGAATGAAGAAAATACCCAGCACCCTTCAAGAGAGGGTGCTGGGTATTTTCTTCATTCTTTTTTTG  AATTCAAAAAAAGAATGAAGAAAATACCCAGCACCCTCTCTTGAAGGGTGCTGGGTATTTTCTTCATTCTCG |
| CircFAM13B shRNA-NC top  CircFAM13B shRNA-NC bottom | GATCCGTTCTCCGAACGTGTCACGTAATTCAAGAGATTACGTGACACGTTCGGAGAATTTTTTC  AATTGAAAAAATTCTCCGAACGTGTCACGTAATCTCTTGAATTACGTGACACGTTCGGAGAACG |
| PKM2 siRNA-1 sense  PKM2 siRNA-1 anti-sense | GGCUGGACUACAAGAACAUTT  AUGUUCUUGUAGUCCAGCCTT |
| PKM2 siRNA-2 sense  PKM2 siRNA-2 anti-sense | GCCAUCUACCACUUGCAAUTT  AUUGCAAGUGGUAGAUGGCTT |
| IGF2BP1 siRNA-1 sense  IGF2BP1 siRNA-1 anti-sense | GCAAGCUAUCAUGAAGCUATT  UAGCUUCAUGAUAGCUUGCTT |
| IGF2BP1 siRNA-2 sense  IGF2BP1 siRNA-2 anti-sense | CCGCGUGCAAGAUGAUCUUTT  AAGAUCAUCUUGCACGCGGTT |
| HNRNPL siRNA-1 sense  HNRNPL siRNA-1 anti-sense | CACGCUUGAAUGUGUUCAATT  UUGAACACAUUCAAGCGUGTT |
| HNRNPL siRNA-2 sense  HNRNPL siRNA-2 anti-sense | GCAGCCGACAACCAAAUAUTT  AUAUUUGGUUGUCGGCUGCTT |
| ADAR1 siRNA-1 sense  ADAR1 siRNA-1 anti-sense | GGACUCCGAAGAAAGAAAUTT  AUUUCUUUCUUCGGAGUCCTT |
| ADAR1 siRNA-2 sense  ADAR1 siRNA-2 anti-sense | GAGGCUUCAUCAGGUUUCUTT  AGAAACCUGAUGAAGCCUCTT |
