# Supplementary material for: HNRNPL induced circFAM13B increased bladder cancer immunotherapy sensitivity via inhibiting glycolysis through IGF2BP1/PKM2 pathway
Source: J Exp Clin Cancer Res. 2023 Feb 6;42:41. doi: 10.1186/s13046-023-02614-3 (PMC9901087; doi:10.1186/s13046-023-02614-3)
Supplement: Supplementary file 2 — Additional file 2: Table S2. All PCR primers used in this research. [file 13046_2023_2614_MOESM2_ESM.doc]

**Additional file 2: Table S2:** All PCR primers used in this research.

| **Primers and probes** |  | **sequence** |
| --- | --- | --- |
| CircFAM13B | Forward  Reverse | 5’-ATGCCAGTGAAAGTAACAGAGACTG-3’  5’-TGTTCCACAGCTGCTCTAATTG-3’ |
| FAM13B  Pre-FAM13B mRNA | Forward  Reverse  Forward  Reverse | 5’-GCAGGGAGGACATCCAGACAATGAG-3’  5’-TGTCGTATCTCTGCCGAAGCCACTC-3’  5’-CAAGTGAACCTCCAGCTTCG-3’  5’-CCCTCTTGGTCTGACACACA-3’ |
| IGF2BP1  PKM2  Hnrnpl  U2  β-Actin | Forward  Reverse  Forward  Reverse  Forward  Reverse  Forward  Reverse  Forward  Reverse | 5’-GCGGCCAGTTCTTGGTCAA-3’  5’-TTGGGCACCGAATGTTCAATC-3’  5’-ATGTCGAAGCCCCATAGTGAA-3’  5’-TGGGTGGTGAATCAATGTCCA-3’  5’-TACGCAGCCGACAACCAAATA-3’  5’-CTCCGGGAGTCATCCGAGT-3’  5’-CATCGCTTCTCGGCCTTTTG-3’  5’-TGGAGGTACTGCAATACCAGG-3’  5’-CTCCATCCTGGCCTCGCTGT-3’  5’-GCTGTCACCTTCACCGTTCC-3’ |
