# Supplementary figures and images for: HNRNPL induced circFAM13B increased bladder cancer immunotherapy sensitivity via inhibiting glycolysis through IGF2BP1/PKM2 pathway
Source: J Exp Clin Cancer Res. 2023 Feb 6;42:41. doi: 10.1186/s13046-023-02614-3 (PMC9901087; doi:10.1186/s13046-023-02614-3)

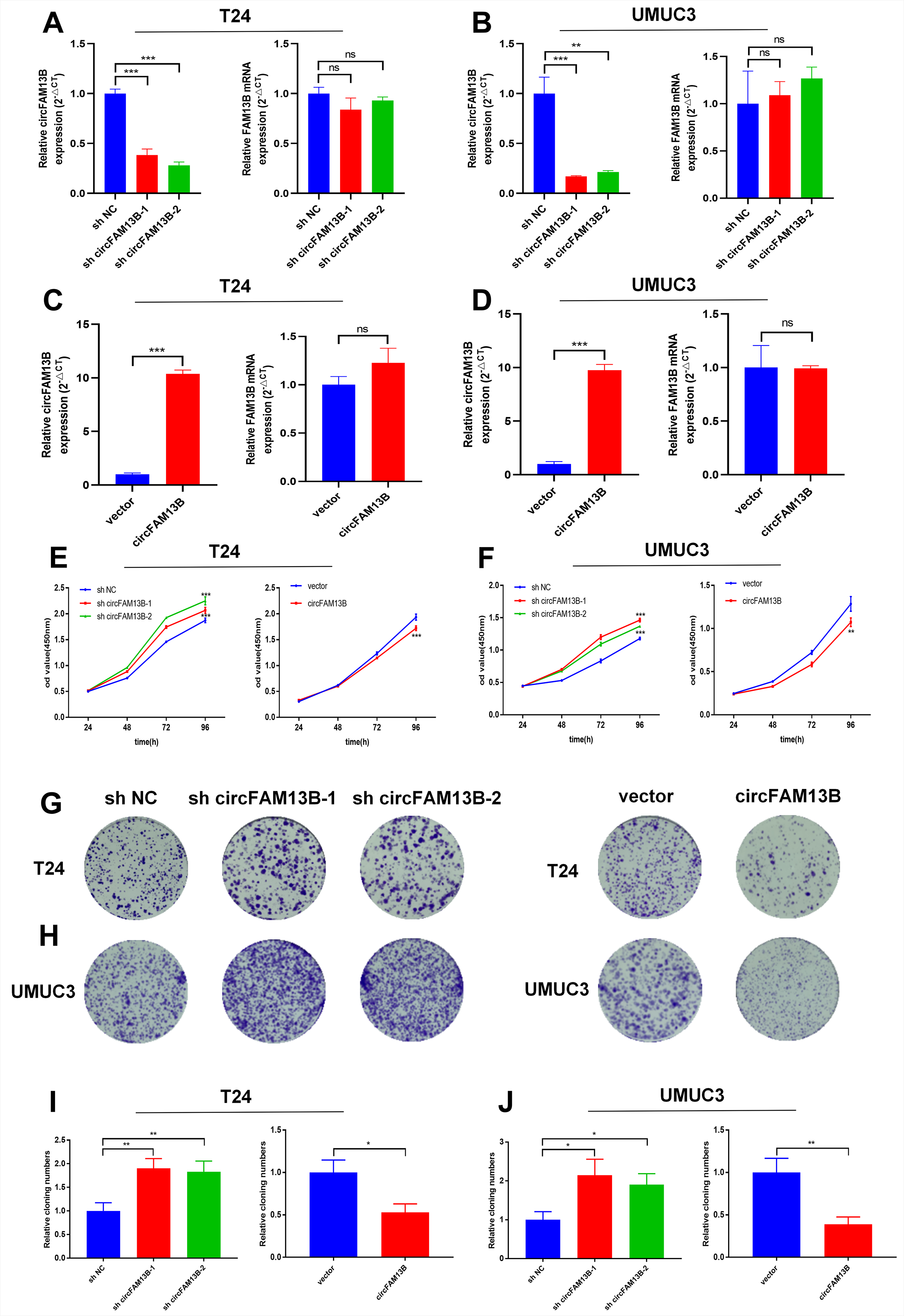

Supplement: Supplementary file 3 — Additional file 3: Figure S1. CircFAM13B inhibited the proliferation of BCa cells. A-B. The efficiency of circFAM13B knockdown in T24 and UMUC3 cells was verified by qRT-PCR (**P<0.01, ***P<0.001, Student’s t-test). C–D. The efficiency of circFAM13B overexpression in T24 and UMUC3 cells was verified by qRT-PCR (***P<0.001, Student’s t-test). E. CCK8 assays showed that circFAM13B inhibited the proliferation of T24 cells (***P<0.001, Student’s t-test). F. CCK8 assays showed circFAM13B inhibited the proliferation of UMUC3 cells (**P<0.01, ***P<0.001, Student’s t-test). G. Colony formation assays confirmed circFAM13B inhibited the proliferation of T24 cells. H. Colony formation assays confirmed that circFAM13B inhibited the proliferation of UMUC3 cells. I–J. Histograms of colony formation assays (*P<0.05, **P<0.01, Student’s t-test). Data are expressed as mean±SD, n=3. [file 13046_2023_2614_MOESM3_ESM.tif]

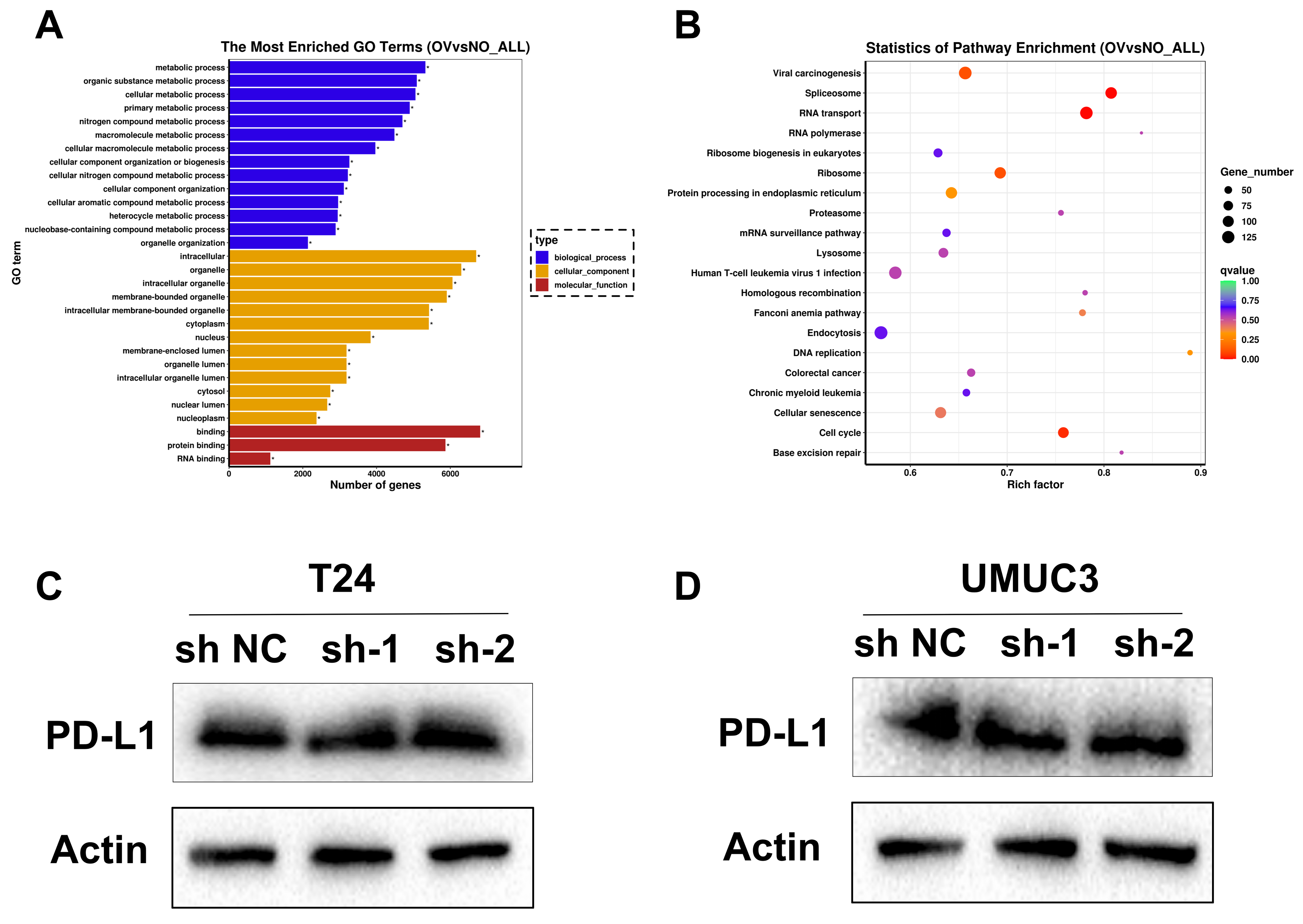

Supplement: Supplementary file 4 — Additional file 4: Figure S2. GO and KEGG analysis of circFAM13B relative mRNA sequencing. A. GO analysis was conducted on the differentially expressed genes of mRNA sequencing. B. KEGG analysis was conducted on the differentially expressed genes of mRNA sequencing. C. Western blot was conducted to investigate the influence of circFAM13B on PD-L1 in T24 cells. D. Western blot was conducted to investigate the influence of circFAM13B on PD-L1 in UMUC3 cells. [file 13046_2023_2614_MOESM4_ESM.png]

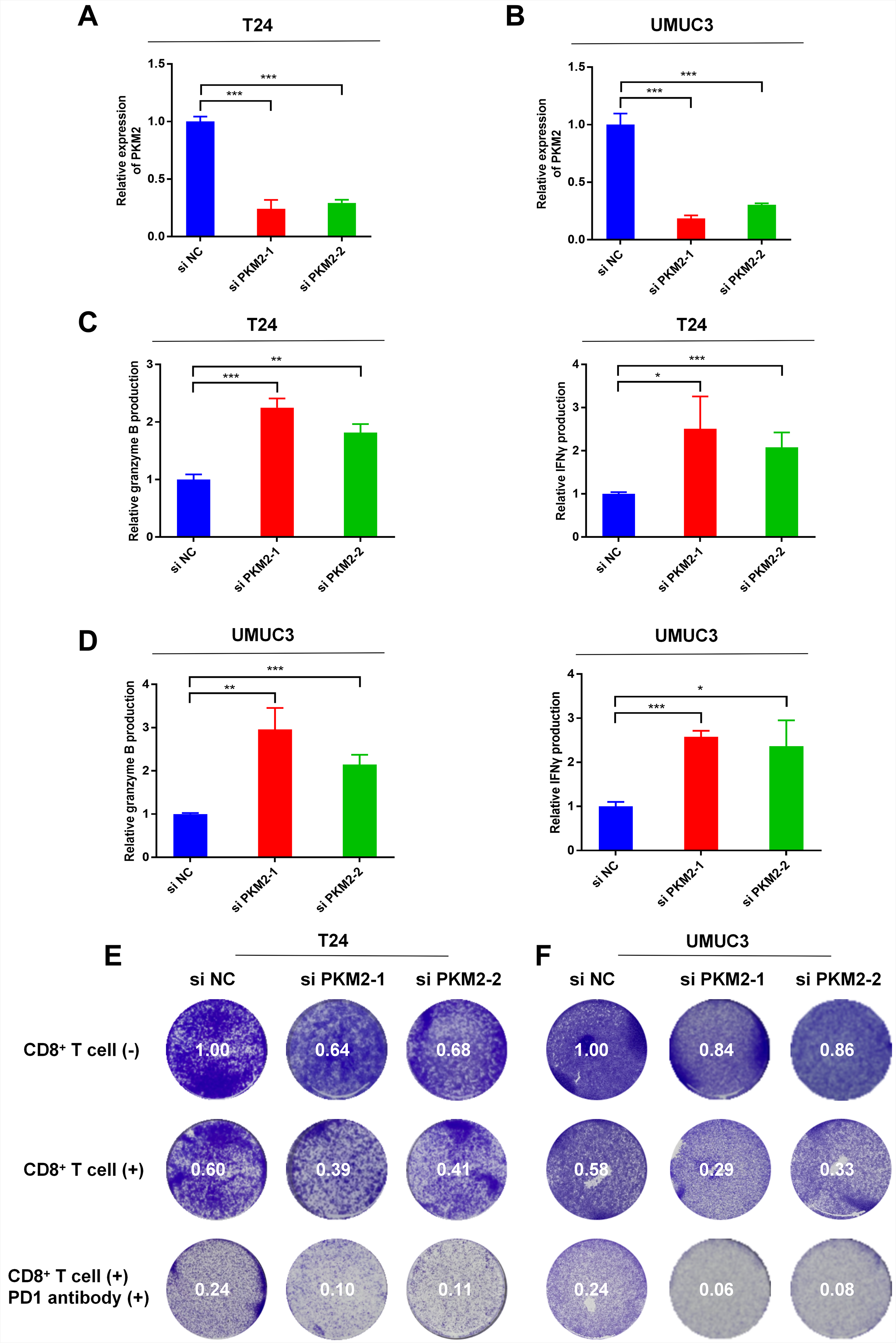

Supplement: Supplementary file 5 — Additional file 5: Figure S3. PKM2 knockdown inhibited the immune escape of BCa cells. A-B. QRT-PCR assays were performed to validate the efficiency of PKM2 siRNAs transfection in T24 and UMUC3 cells (***P<0.001, Student’s t-test). C–D. ELISA assays were carried out to detect the granzyme B and IFN-γ produced by CD8+ T cells, which were co-cultured with PKM2 siRNAs transfected or control T24 and UMUC3 cells (*P<0.05, **P<0.01, ***P<0.001, Student’s t-test). E–F. The killing ability of CD8+ T cells and the immunotherapy sensitivity of BCa were increased after being co-cultured with PKM2 knockdown T24 or UMUC3 cells. Data are expressed as mean±SD, n=3. [file 13046_2023_2614_MOESM5_ESM.tif]

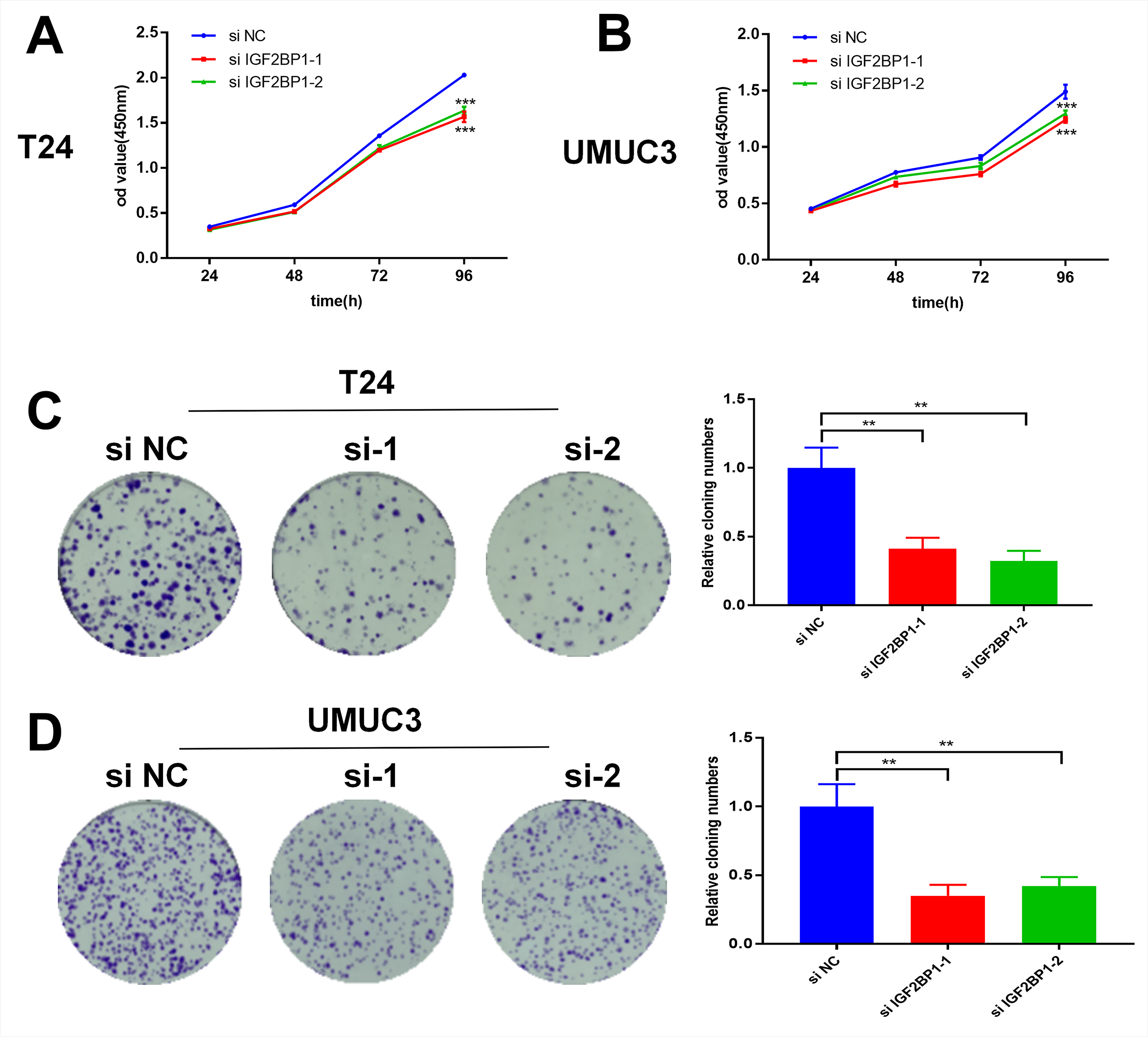

Supplement: Supplementary file 6 — Additional file 6: Figure S4. IGF2BP1 promoted the proliferation of BCa cells. A-B. CCK8 assays were carried out in IGF2BP1 siRNAs transfected or control T24 and UMUC3 cells (***P<0.001, Student’s t-test). C–D. Colony formation assays were performed in IGF2BP1 siRNAs transfected or control T24 and UMUC3 cells (**P<0.01, Student’s t-test). Data are expressed as mean±SD, n=3. [file 13046_2023_2614_MOESM6_ESM.tif]

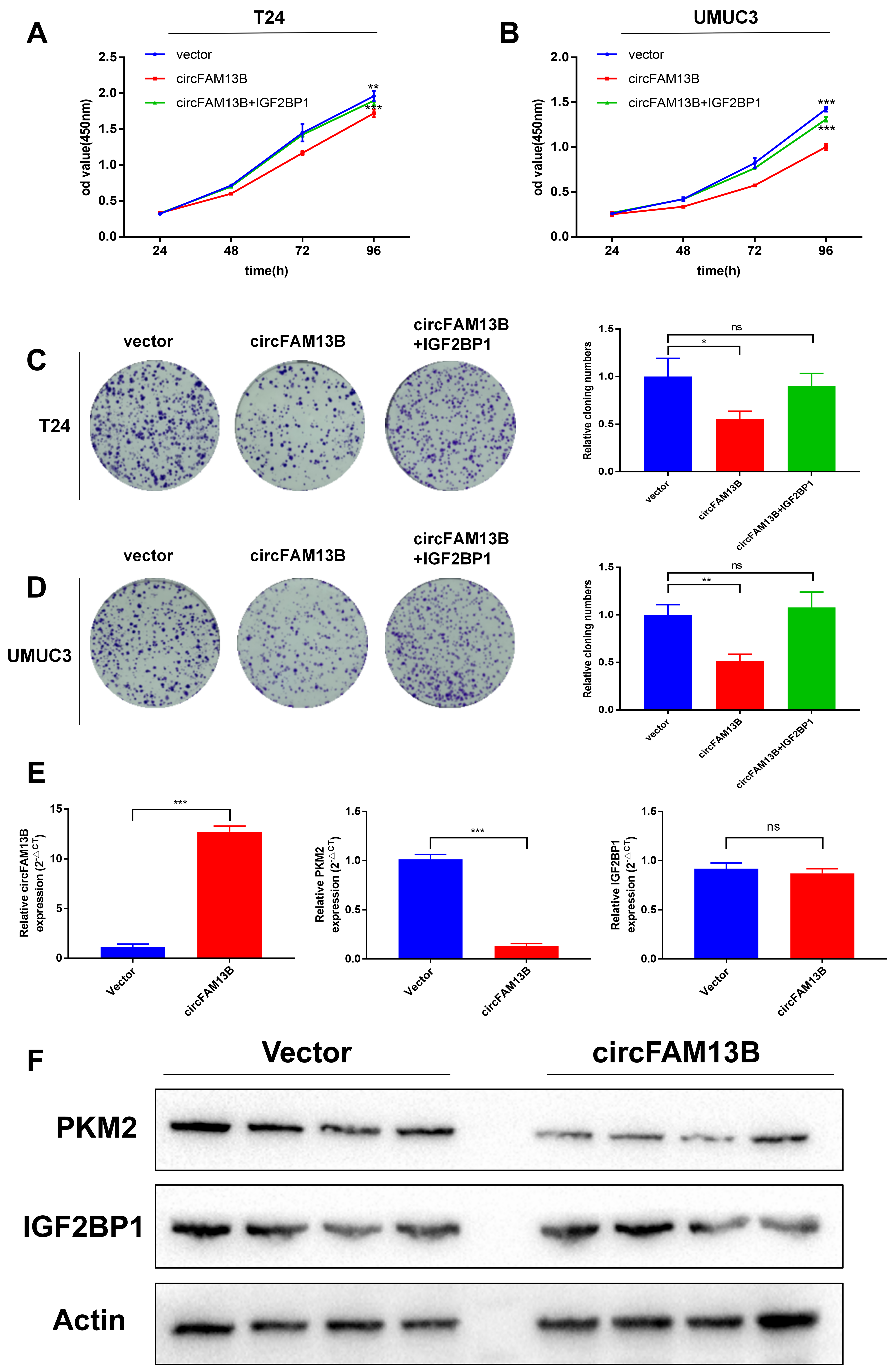

Supplement: Supplementary file 7 — Additional file 7: Figure S5. IGF2BP1 overexpression rescued the repressed proliferation induced by circFAM13B in BCa. A-B. CCK8 assays showed that the overexpression of IGF2BP1 rescued the inhibition of proliferation caused by circFAM13B in T24 and UMUC3 cells (**P<0.01, ***P<0.001, Student’s t-test). C–D. Colony formation assays showed that the overexpression of IGF2BP1 rescued the inhibition of proliferation caused by circFAM13B in T24 and UMUC3 cells (*P<0.05, **P<0.01, Student’s t-test). E. qRT-PCR confirmed circFAM13B inhibited PKM2 expression in tumors of NOG mice without influencing the expression of IGF2BP1 (***P<0.001, Student’s t-test). F. Western blot confirmed circFAM13B inhibited PKM2 expression in tumors of NOG mice without influencing the expression of IGF2BP1. Data are expressed as mean±SD, n=3. [file 13046_2023_2614_MOESM7_ESM.png]

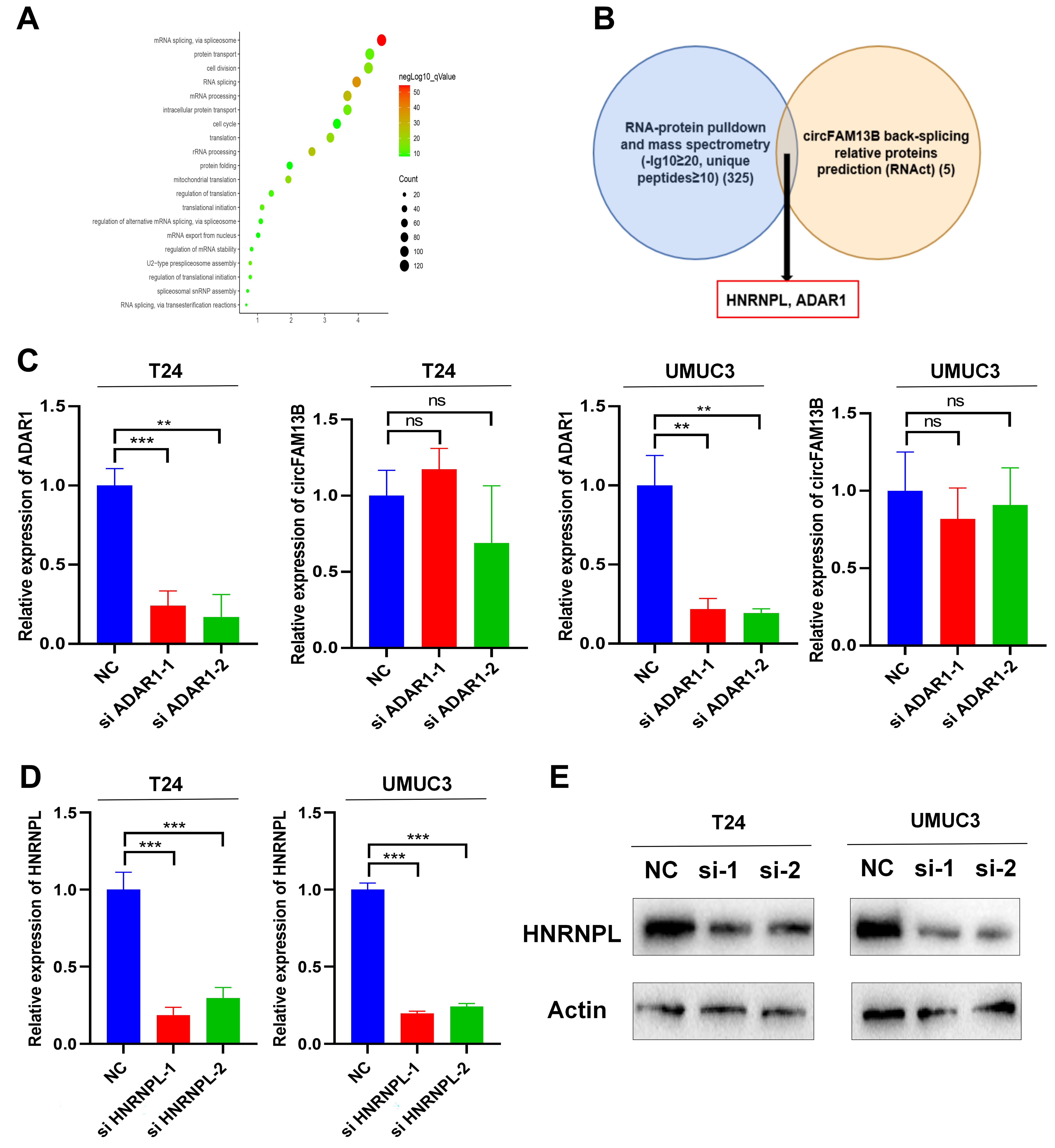

Supplement: Supplementary file 8 — Additional file 8: Figure S6. Construction of HNRNPL knockdown T24 and UMUC3 cells. A. GO analysis was conducted on the proteins pulled down by flanking introns probe. B. The results of RNAct database predictions and circFAM13B flanking introns relative mass spectrometry analysis were intersected, and HNRNPL and ADAR1 were found. C. The expression of ADAR1 and circFAM13B in T24 or UMUC3 cells were confirmed by qRT-PCR after ADAR1 siRNA transfection (**P<0.01, ***P<0.001, Student’s t-test). D. The knockdown efficiency of HNRNPL siRNAs transfection in T24 and UMUC3 cells were confirmed by qRT-PCR (***P<0.001, Student’s t-test). E. The knockdown efficiency of HNRNPL siRNAs transfection in T24 and UMUC3 cells were confirmed by Western blot analysis. Data are expressed as mean±SD, n=3. [file 13046_2023_2614_MOESM8_ESM.png]
